# Intercostal Nerve Cryoablation Therapy in Thoracic and Cardiac Surgery for Postoperative Pain Management: A Systematic Review and Meta-Analysis

**DOI:** 10.1093/icvts/ivag143

**Published:** 2026-05-23

**Authors:** Christopher W Towe, Zachary M Bauman, Lizabeth A O’Connor, Curtis C Quinn, Marc P Pelletier, Alyssa K Hahn, Nfii Ndikintum, Madelyn M Dua, Edward Cantu

**Affiliations:** Department of Surgery, Division of Thoracic and Esophageal Surgery, University Hospitals Cleveland Medical Center, Cleveland, OH 44106, United States; Department of Surgery, Division of Acute Care Surgery, University of Nebraska Medical Center, Omaha, NE 68198, United States; Division of Thoracic Surgery, Elliot Health System, Manchester, MA 03103, United States; Division of Thoracic Surgery, Elliot Health System, Manchester, MA 03103, United States; Division of Cardiac Surgery, Yale New Haven Hospital, New Haven, CT 06520, United States; AtriCure, Inc, Mason, OH 45040, United States; AtriCure, Inc, Mason, OH 45040, United States; AtriCure, Inc, Mason, OH 45040, United States; Department of Surgery, Division of Cardiovascular Surgery, Hospital of the University of Pennsylvania, Philadelphia, PA 19104, United States

**Keywords:** intercostal nerve cryoablation, cardiac surgery, thoracic surgery, hospital length of stay, opioid consumption, meta-analysis, pain management

## Abstract

**Objectives:**

Thoracic and cardiac surgical procedures are associated with significant postoperative pain. Intercostal nerve cryoablation (INC) is a non-opioid adjunctive pain management strategy. The objective of this study was to comprehensively review published outcomes of INC during non-pectus repair thoracic and cardiac surgeries to inform clinical practice and guideline development.

**Methods:**

A literature search was conducted in PubMed, Embase, Google Scholar, and using manual approaches to identify comparative studies of patients undergoing non-pectus repair thoracic or cardiac procedures with INC versus standard of care (SOC) without INC. Meta-analyses were performed to quantitively evaluate opioid consumption and hospital length of stay (LOS). Secondary outcomes were summarized qualitatively.

**Results:**

Twenty-four studies were included encompassing 18465 patients, of whom 10.6% (*n* = 1954) received INC. INC was applied during surgical stabilization of rib fractures, thoracotomy, pulmonary resections, lung transplants, aortic aneurysm repair, and cardiac procedures. Meta-analyses of adult studies demonstrated a significant reduction in inpatient opioid consumption by 102 morphine milligram equivalents (MME) (95% CI: −180.00, −23.87) and a non-significant reduction in opioid consumption after discharge by 89 MME (95% CI: −182.00, 4.56) with INC. Sub-group analysis demonstrated the largest effect size in inpatient opioid reduction for bilateral thoracotomy or thoracosternotomy for lung transplants. Meta-analysis demonstrated no significant difference in hospital stay for adult patients treated with INC.

**Conclusions:**

The results of this systematic review and meta-analysis provide evidence to support the association between INC and reduced inpatient opioid consumption in non-pectus repair thoracic and cardiac procedures.

## Graphical Abstract

**Figure:**
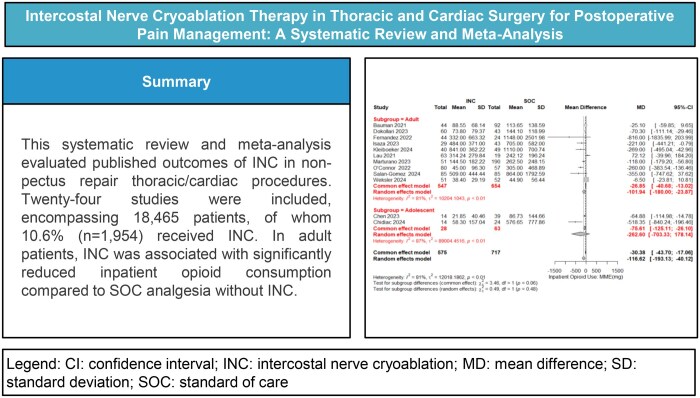

## INTRODUCTION

Thoracic and cardiac surgical procedures are associated with significant postoperative pain. Current pain management strategies include epidural analgesia, patient-controlled analgesia, intercostal nerve blocks, paravertebral regional blocks, and other multimodal regimens incorporating intravenous and oral opioids, nonsteroidal anti-inflammatory drugs, acetaminophen, and/or gabapentinoids.^1^^,^^2^ However, many interventions are limited by a short therapeutic window and adverse systemic effects.^1^^,^^2^ Additionally, pain management has shifted towards opioid-sparing regimens due to the opioid epidemic and risk of dependence.^3–6^

Intercostal nerve cryoablation (INC) is used as a non-opioid adjunct for pain management following thoracic and cardiac surgery. INC applies extreme cold (approximately −70°C) via a probe to extract heat from the tissue and destroy nerve axons, leaving the perineural connective tissue intact. This disrupts sensory neural pathways from propagating pain signals until axon regeneration several weeks to months later, providing prolonged duration analgesia.^7^ Current cryoprobes generate rapid cooling via the Joule-Thomson expansion of nitrous oxide or carbon dioxide to ablate the nerves and achieve this long-lasting conduction block. INC is advantageous because it is not associated with some of the adverse systemic effects of other analgesic techniques, with demonstrated safety, efficacy, and longer duration of pain relief.^7^

The clinical outcomes of INC are most widely documented in pectus excavatum repair (ie, Nuss procedure), as documented in prospective and retrospective studies, systematic reviews, and meta-analyses.^8–10^ In pectus repair procedures, hospital length of stay (LOS) is mostly driven by adequate pain management.^11^ In these procedures, INC provides adequate adjunctive pain management and significant reductions in LOS and opioid consumption.^8^^,^^10^ INC has also shown promising results in other thoracic and cardiac procedures. However, to our knowledge, no comprehensive review exists that synthesizes published evidence on the outcomes of INC in non-pectus repair thoracic and cardiac surgeries. The goal of this study was to systematically review published literature on the use of INC during non-pectus repair thoracic and cardiac surgeries for qualitative and quantitative analyses.

## METHODS

Prior to the initiation of the search, a protocol was developed in compliance with the Preferred Reporting Items for Systematic reviews and Meta-Analyses (PRISMA) statement. The protocol was not registered in a public domain. This article is based on previously conducted studies and does not contain any new studies with human participants or animals performed by any of the authors.

### Data sources and search criteria

A systematic search was performed across 3 databases: PubMed (via the National Library of Medicine), Embase, and Google Scholar. A manual search was subsequently performed to identify other non-indexed but potentially relevant articles. The search was limited to articles available in English published in the last 10 years (2014-2024). The database searches were conducted on May 13, 2024 (Embase), December 17, 2024 (PubMed), and February 5, 2025 (Google Scholar).

The search was limited to comparative studies of patients undergoing thoracic or cardiac procedures with INC versus standard of care (SOC) analgesia without INC, with a measure of at least 1 of the following end-points post-index procedure: hospital LOS, opioid consumption, hospital cost and/or charges, complications, readmission, and/or emergency department (ED) visits. The SOC comparator was defined as perioperative analgesia without INC, recognizing that specific analgesic strategies varied by institution. Exclusion criteria included percutaneous cryoablation, cryosurgery, repair of pectus excavatum as the index procedure, conference abstracts and presentations, videos, preclinical studies, case reports, editorials/commentaries, review articles, meta-analyses, and studies with known overlapping patient populations. The following keywords were used to develop search strings for database queries: (i) patient population: cardiac, thoracic, thorax, heart, chest, lung, rib; (ii) intervention: cryoablation, cryoneurolysis, cryoanalgesia, cryo, nerve block, intercostal nerve, peripheral nerve; (iii) device: AtriCure, cryoNB, cryoICE, and cryoSPHERE. These keyword groups were combined using Boolean operators to construct database-specific search strings, which are provided in full in **Table S1**.

### Study screening and selection

Initial screening of titles and abstracts of all records from the database and manual searches was conducted by 2 authors (A.H., M.D.). Duplicate records and those clearly ineligible were excluded from further review. The full texts were reviewed for the remaining articles and assessed for final eligibility. If disagreements arose and could not be resolved between these 2 reviewers, additional authors would have been consulted, and a vote would have been taken to determine inclusion. However, in this review, all discrepancies were resolved through discussion between the 2 screeners, and no additional authors were required. Although dedicated screening software was not used, the dual-reviewer process and predefined criteria were designed to ensure accuracy and reproducibility in study selection.

### Data extraction

Data on the following variables were extracted from articles selected for inclusion: study design, country, procedure type, treatment arms, patient population characteristics (*n*, age, sex, body mass index [BMI], race), intervention characteristics (cryoablation device, cryoablation temperature, duration of freeze, number and level of intercostal nerves ablated), and outcomes measures (hospital LOS, intensive care unit [ICU] LOS, inpatient and outpatient opioid consumption, opioid prescriptions, hospital cost/charges, postoperative pulmonary function, complication rates, 30-day readmission, reoperation, and ED visits). Complication rates were compiled across all studies for neuropathic symptoms, complications commonly related to opioid use, and pulmonary complications.

### Quantitative and qualitative data analysis

Inpatient and outpatient opioid consumption and hospital LOS were quantitatively analysed by calculating the weighted mean difference between INC and SOC cohorts. Inpatient opioid consumption was defined as opioid use during inpatient hospital stay. Outpatient opioid consumption was defined as opioid prescriptions at discharge or opioid use after discharge. To help standardize data and units for quantitative pooling, several assumptions were made as follows. If the mean and/or SD were not reported, the median was used in place of the mean and SD was estimated using interquartile ranges (IQR). Values not reported in tables or text were estimated from figures. Additionally, units for opioid consumption (eg, morphine milligram equivalents [MME]/kg, MME/day) were standardized to MME (mg) for quantitative assessment, if possible. If units of opioid consumption could not be estimated in MME, the study was excluded from quantitative analysis for opioid consumption.

Meta-analyses were performed in R version 4.41 using common effect and random effects models weighted by inverse variance, using forest plots for visualization of results. Heterogeneity was assessed across studies using the DerSimonian-Laird method (*I*^2^) test for heterogeneity, with a *P-*value less than .05 indicative of significant heterogeneity. The random effects model is a more conservative estimate employed when significant heterogeneity is present. Studies that did not report variance were not included in the meta-analyses. If needed, sensitivity analyses were performed. Studies with suspected overlap in patient populations (eg, database studies) were excluded from sensitivity analyses. Sub-group analyses were performed to explore the effect of INC in different surgical approaches and patient populations; however, formal tests for sub-group differences were not performed, as the analyses were intended to explore heterogeneity rather than to formally compare effects between subgroups. Studies with mixed surgical approaches were excluded from sub-group analyses. At least 2 studies were required per sub-group to carry out a meta-analysis.

Other outcomes including % change in opioid consumption, opioid prescriptions, hospital costs/charges, postoperative pulmonary function, and complications were qualitatively analysed because of inconsistent reporting and/or substantial variability in reporting methods across studies. To demonstrate how opioid consumption changed with INC, the % change in opioid consumption was calculated by comparing the reported INC cohort opioid consumption relative to the SOC cohort opioid consumption. Raw values reported by study authors were used for calculating the % change in opioid consumption. For qualitative analysis of hospital costs/charges and measures of postoperative pulmonary function, the mean (SD) or median (IQR) was reported for the INC and SOC cohorts with a measure of statistical significance, if reported. For complications and the proportion of patients discharged with an opioid prescription or using opioids at outpatient visits, the reported rates for the INC and SOC cohorts were assessed, with a measure of statistical significance, if reported.

### Quality and risk-of-bias assessment

Two authors (A.H., M.D.) independently assessed the methodological quality of included studies using the Cochrane Risk-of-Bias Tool for randomized controlled trials (RCTs) and the Newcastle-Ottawa Quality Assessment Scale for Cohort Studies. Funnel plots and Egger’s regression test for small-study effects were performed to assess publication bias.

## RESULTS

### Study selection

**Figure 1.**
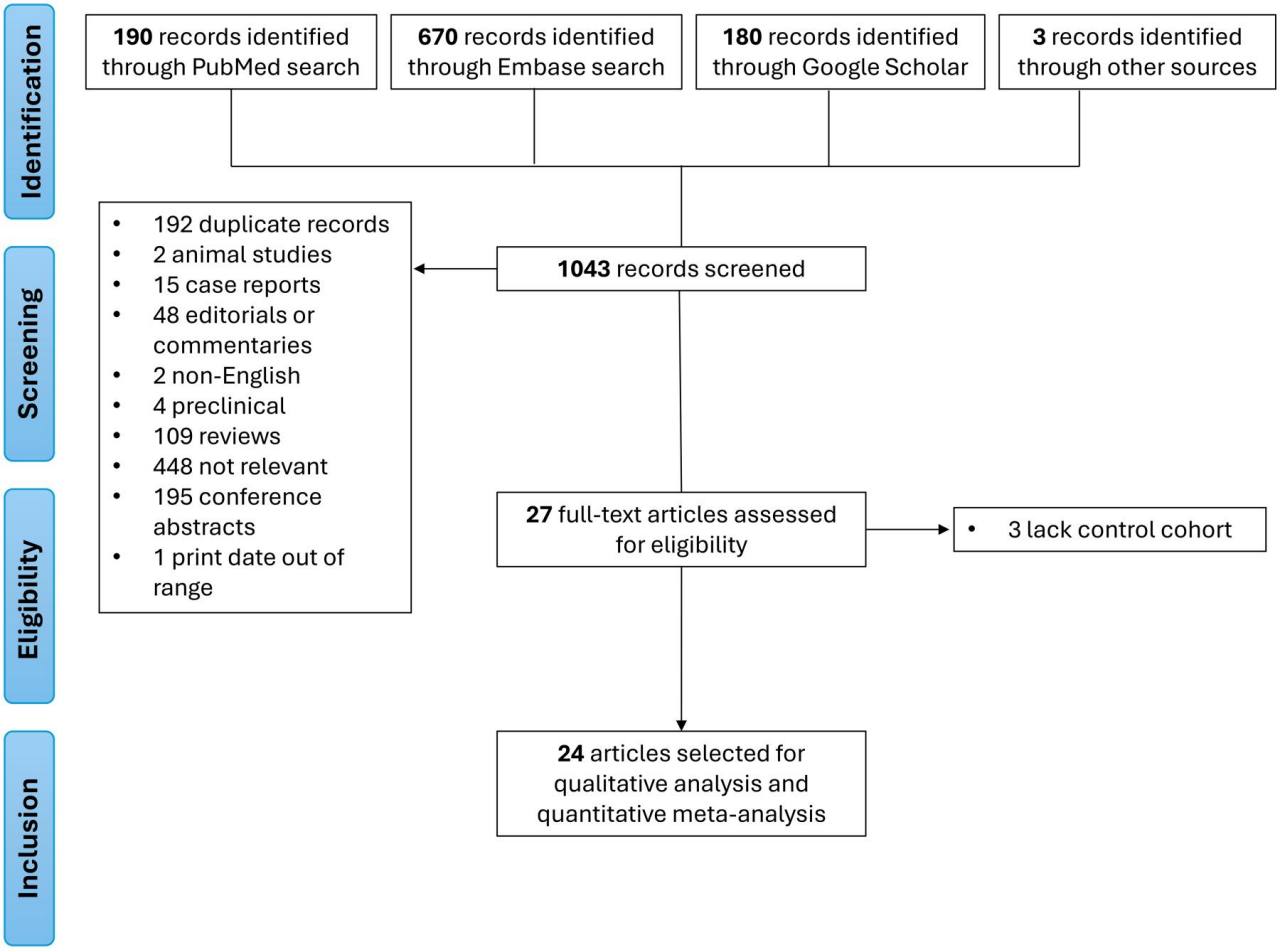
PRISMA Flow Chart

The search strategy yielded 1043 articles (PubMed: 190; Embase: 670; Google Scholar: 180; manual search: 3) for review. After initial screening of titles and abstracts, 1016 were removed from further review. Full text article review of the remaining 27 articles resulted in exclusion of 3 additional articles. The remaining 24 articles were selected for inclusion (**Figure 1**).^12–35^

### Study characteristics

Of the 24 included studies, there were 17 single-center^13^^,^^14^^,^^16–27^^,^^31^^,^^32^^,^^34^ and 4 multicentre retrospective observational studies,^12^^,^^15^^,^^29^^,^^35^ 1 multicentre prospective RCT,^28^ and 2 single-center prospective RCTs.^30^^,^^33^ INC was applied during various surgical procedures including surgical stabilization of rib fractures (SSRF),^12–16^^,^^29^ thoracotomy for paediatric/adolescent cancer-related indication,^17–19^ cardiac procedures including isolated coronary artery bypass grafting^27^ and minimally invasive heart valve surgery,^28^ aortic aneurysm repair,^25^^,^^26^ thoracotomies for pulmonary pathologies (lung resection, lobectomy, decortication),^20^^,^^21^^,^^24^^,^^30^^,^^31^^,^^33^^,^^35^ lung transplants,^22–24^^,^^32^^,^^34^ and transthoracic hiatal hernia repair (TTHHR)^24^ (**Table 1**). Overall, there was a low risk-of-bias in RCTs and good study quality for observational studies, as shown by scores ranging from 6 to 9 on the Newcastle-Ottawa scale. Risk-of-bias assessments are shown in **Tables S2 and S3**.

**Table 1.** Study Characteristics and Demographic Information

Study	Procedure	Country	Study design	Treatment arm	Participants, *n*	Male, *n* (%)	Age (years)	BMI (kg/m^2^)	Race: White
Bauman et al 2021 *Injury*	Surgical stabilization of rib fractures	United States	Multicentre retrospective observational study	INC	44	29 (65.90%)	58.86 (13.64)	NR	NR
				SOC	92	68 (73.90%)	56.76 (15.66)	NR	NR
Choi et al 2021 *J Trauma Acute Care Surg*	Surgical stabilization of rib fractures	United States	Single-center retrospective observational study	INC	20	16 (80%)	55 (45.0-67.3)	NR	NR
				SOC	14	12 (86%)	55 (37.0-60.8)	NR	NR
Fernandez et al 2022 *J Trauma Acute Care Surg*	Surgical stabilization of rib fractures	United States	Single-center retrospective observational study	INC	44	31 (70.5%)	59 (52-69)	27.3 (24.9-30.0)	37 (84.1%)
				SOC	24	20 (83.3%)	57 (50-68)	26.8 (25.8-32.3)	23 (95.8%)
Marturano et al 2023 *Injury*	Surgical stabilization of rib fractures	United States	Multicentre retrospective observational study	INC	51	38 (74.5%)	60 (51.0-70.0)*	NR	NR
				SOC	190	140 (73.7%)	55 (42.0-65.0)*	NR	NR
O’Connor et al 2023 *Injury*	Surgical stabilization of rib fractures	United States	Single-center retrospective observational study	INC	14	11 (78%)	54 (12.3)	30.8 (7.2)	NR
				SOC	12	7 (58%)	52 (20.5)	33.1 (8.8)	NR
Aryan et al 2024 *J Surg Res*	Surgical stabilization of rib fractures	United States	Multicentre retrospective observational study	INC	750	541 (72.1%)	59 (47, 67)	NR	NR
				SOC	15,034	11,089 (73.9%)	57 (46, 67)	NR	NR
Chen et al 2023 *J Surg Res*	Thoracotomy for paediatric/adolescent cancer-related indication	United States	Single-center retrospective observational study	INC	8	4 (50%)	13.5 (12-15)	NR	2 (25%)
				SOC	24	15 (62.5%)	15 (11-18)	NR	6 (25%)
Chidiac et al 2024 *J Surg Res*	Thoracotomy for paediatric/adolescent cancer-related indication	United States	Single-center retrospective observational study	INC	14	9 (64.3%)	17 (14-18.5)	18.0 (17.2-24.4)	NR
				SOC	24	19 (79.2%)	17 (16-18.5)	20.5 (18.8-24.0)	NR
McElhinney et al 2024 *Pediatr Blood Cancer*	Thoracotomy for paediatric/adolescent cancer-related indication	United States	Single-center retrospective observational study	INC	23	11 (47.8%)	16 (14.5-17)	NR	12 (52.2%)
				SOC	15	6 (40%)	16 (13-16)	NR	12 (80%)
Ba et al 2015 *Surg Today*	Lobectomy by open thoracotomy	China	Single-center prospective, randomized clinical trial	INC	87	56 (64.4%)	68.2 (41-78)	NR	NR
				SOC	91	59 (64.8%)	66.8 (45-77)	NR	NR
Maxwell et al 2023 *Innovations*	Minimally invasive pulmonary resection	United States	Single-center retrospective observational study	INC	23	7 (30.4%)	72.96 (9.42)	26.27 (4.55)	NR
				SOC	26	8 (30.8%)	68.42 (11.03)	27.94 (7.21)	NR
Miller et al 2024 *Pain Ther*	Single surgical lobectomy	United States	Multicentre retrospective observational study of national data	INC	266	120 (45.1%)	68.6 (10.1)	NR	181 (68.0%)
				SOC	266	123 (46.2%)	68.8 (9.5)	NR	201 (75.6%)
O’Connor et al 2022 *J Surg Res*	Unilateral thoracic surgery for pulmonary pathologies (wedge resection, lobectomy, decortication)	United States	Single-center retrospective observational study	INC	80	46 (57.5%)	61.1 (15.1)	28.64 (8.4)	NR
				SOC	57	33 (57.8%)	61.3 (16)	28.53 (8.1)	NR
Tung et al 2022 *J Robot Surg*	Robotic-assisted pulmonary resections	United States	Single-center retrospective observational study	INC	10	NR	69.4 (8.1)	NR	NR
				SOC	10	NR	67.4 (6.1)	NR	NR
Isaza et al 2023 *Pain Ther*	Bilateral lung transplantation via clamshell incision	United States	Single-center retrospective observational study	INC	29	19 (65.5%)	66 (63-69)*	NR	18 (62.1%)
				SOC	43	24 (55.8%)	58 (49-65)*	NR	29 (67.4%)
Koons et al 2023 *JTCVS Open*	Single- and double-lung transplantation	United States	Single-center retrospective observational study	INC	45	25 (55.6%)	65 (58-67)	25.3 (22.7-31.9)	38 (84.4%)
				SOC	57	35 (61.4%)	61.4 (49-67)	24.8 (21.5-27.8)	45 (79.0%)
Kleiboeker et al 2024 *JHLT Open*	Single and bilateral lung transplantation	United States	Single-center retrospective observational study	INC	40	29 (72.5%)	60 (56-65)	NR	NR
				SOC	49	33 (67.3%)	62 (48-65)	NR	NR
Pourak et al 2024 *Cardiovasc Thorac Surg*	Transthoracic hiatal hernia repair	United States	Single-center retrospective observational study	INC	33	7 (21.2%)	64.1 (11.6)	NR	33 (100%)
				SOC	43	10 (23.3%)	66.5 (9.8)	NR	40 (93.0%)
	Lung resection			INC	39	23 (59%)	63.4 (10.8)	NR	35 (89.7%)
				SOC	19	12 (63.2%)	59.7 (12.3)	NR	19 (100%)
	Double lung transplantation			INC	20	14 (70.0%)	55.1 (8.7)	NR	18 (90.0%)
				SOC	32	17 (53.1%)	54.8 (9.2)	NR	26 (81.3%)
Salan-Gomez et al 2024 *JTCVS Open*	Lung transplantation	United States	Single-center retrospective observational study	INC	85	53 (62%)	62 (55-68)	26.9 (22.9-31.9)	NR
				SOC	85	56 (66%)	62 (52-67)	25.1 (21.8-30.2)	NR
Weksler et al 2024 *JTCVS*	Minimally invasive thoracic surgery including wedge resection, segmental resection, or lobectomy	United States	Single-center prospective, randomized clinical trial	INC	51	22 (43.1%)	66.2 (61.2-71.6)	28.1 (23.6-32.7)	NR
				SOC	52	20 (38.5%)	70.9 (63.1-75.5)	27 (23.2-32)	NR
Clemence et al 2020 *Semin Thorac Cardiovasc Surg*	Thoracic aortic aneurysm or thoraco-abdominal aortic aneurysm repair	United States	Single-center retrospective observational study	INC	25	22 (88%)	60 (51-67)	NR	NR
				SOC	92	64 (70%)	57 (50-63)	NR	NR
Tanaka et al 2020 *Ann Thorac Surg*	Descending or thoraco-abdominal aortic aneurysm repair	United States	Single-center retrospective observational study	INC	28	21 (75%)	61 (44-70)	NR	NR
				SOC	98	63 (66%)	59 (45-78)	NR	NR
Dokollari et al 2023 *Rev Cardiovasc Med*	Isolated coronary artery bypass grafting	United States	Single-center retrospective observational study	INC	60	47 (78.3%)	69.6 (8.9)	NR	NR
				SOC	43	38 (90.5%)	67.5 (10.1)	NR	NR
Lau et al 2021 *Pain Ther*	Minimally invasive heart valve surgery	United States	Multicentre prospective, randomized clinical trial	INC	65	38 (58%)	63.83 (11.96)	28.61 (5.03)	NR
				SOC	19	11 (58%)	66.42 (12.53)	29.95 (4.73)	NR

Data reported as *n* (%), median (interquartile range), or mean (SD).

* *P-*value < .05 for statistical comparison between cohorts; reported *P-*value based on statistical methods referenced in article.

Abbreviations: INC = intercostal nerve cryoablation; NR = not reported; SOC < standard of care.

A total of 18465 patients were included, of whom 10.6% (*n* = 1954) received INC (**Table 1**). Across all studies, where reported, 71.7% of patients were male, mean ages ranged from 13.5 to 73 years and mean BMIs ranged from 18 to 33.1 kg/m^2^. Of 7 studies (*N* = 1030) that reported race, 75.2% of patients were white.

Twenty-one studies employed the cryoICE cryoablation probes (AtriCure, Inc., Mason, OH) for INC.^12–28^^,^^32–35^ One study employed Frigitronics CE-82 cryoprobes (CooperSurgical, Inc., Trumbull, CT) for INC.^31^ Two studies did not specify the cryoablation device/manufacturer.^29^^,^^30^ Cryoablation temperature ranged from −40 to −80°C across studies, with a freeze duration of 2 min per nerve for most studies. Cryoablation was applied unilaterally or bilaterally to 4 to 7 intercostal nerves at levels ranging from T2 to T10 (**Table S4**). The SOC pain management approach for the SOC without INC group varied widely across studies based on institutional standards, including thoracic epidurals, patient-controlled analgesia, peripheral nerve blocks, multimodal analgesia, elastomeric infusion pumps, opioid and non-opioid medications (**Table S4**).

### Study outcomes

#### Quantitative analysis

##### Opioid consumption

**Figure 2.**
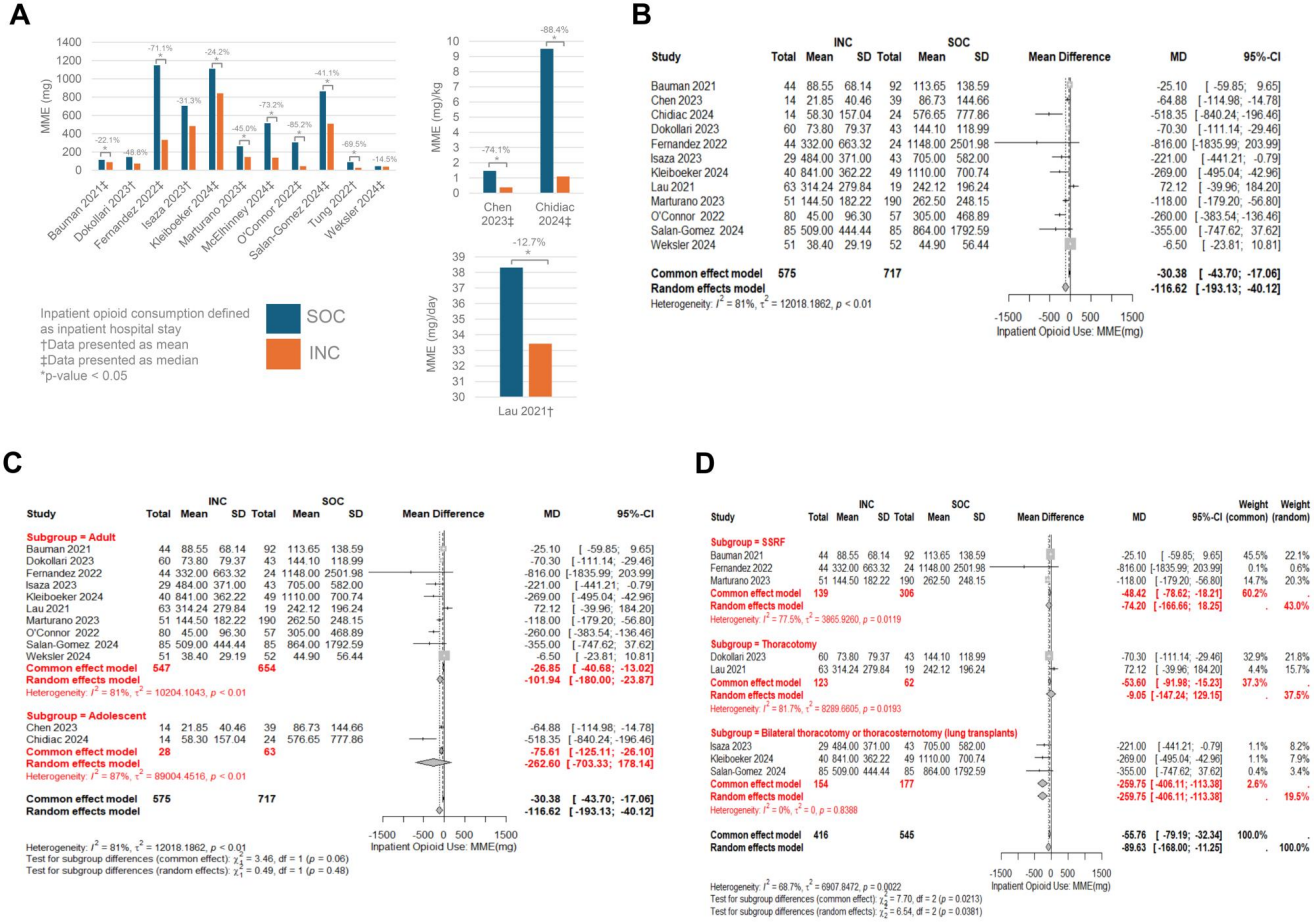
Inpatient opioid consumption. (**A)** Inpatient opioid consumption for INC and SOC cohorts. Percent change in inpatient opioid consumption with INC is reported above each cohort comparison (*P-value < .05). (**B**) Forest plot of inpatient opioid consumption. (**C**) Sub-group analyses by patient population age groups. (**D**) Sub-group analyses by surgical approach. Meta-analyses for each individual sub-group are presented in red. The overall pooled results for the common effect and random effects models for all studies are presented in black at the bottom of the panel. INC: intercostal nerve cryoablation; MD: mean difference; MME: morphine milligram equivalents; SOC: standard of care; SSRF: surgical stabilization of rib fractures

Fifteen studies^12^^,^^14–19^^,^^21^^,^^22^^,^^27^^,^^28^^,^^31–34^ reported inpatient opioid use, with 11 studies^12^^,^^14–19^^,^^21^^,^^31^^,^^32^^,^^34^ individually reporting significantly reduced opioid consumption in the INC cohort compared to the SOC. The % reduction in opioid consumption across studies ranged from 12.7% to 88.4% (**Figure 2A**). In an overall pooled analysis spanning all eligible populations, meta-analysis showed a significant reduction in inpatient opioid consumption with INC by 116.62 MME (95% CI: −193.13, −40.12; **Figure 2B**). However, the corresponding funnel plot exhibited some evidence of asymmetry and Egger’s regression test was statistically significant (*z* = 3.82, *P* = .0001), suggesting potential publication bias or small-study effects (**Figure S1**).

Given the fundamental clinical differences between adult cardiothoracic surgery and paediatric/adolescent oncology surgery, age-based sub-group analyses were performed to allow for clinically relevant interpretation of primary outcomes. When examined separately, adult patients treated with INC experienced a significant reduction in inpatient opioid consumption by 101.94 MME (95% CI: −180.00, −23.87; **Figure 2C**). In contrast, paediatric/adolescent patients demonstrated a reduction in inpatient opioid consumption by 262.60 MME with INC; however, this effect did not reach statistical significance (95% CI: −703.33, 178.14; **Figure 2C**).

Sub-group analyses were carried out to explore the effect of INC on inpatient opioid use by various surgical approaches (**Figure 2D**). Studies that reported inpatient opioid consumption were categorized into the following subgroups based on surgical approach: thoracotomy (regardless of indication), SSRF, or bilateral thoracotomy or thoracosternotomy (lung transplants). Studies that included patient cohorts with mixed surgical approaches were excluded from these subgroups to minimize heterogeneity.^21^ Similarly, studies of paediatric/adolescent patient populations were excluded from the surgical approach sub-group analysis to account for their fundamental difference from adult cardiothoracic surgery and to maintain more homogenous, clinically relevant subgroups.^17^^,^^18^ There was a significant reduction in inpatient opioid consumption for patients treated with INC during bilateral thoracotomy or thoracosternotomy (clamshell approach) for lung transplants by 259.75 MME (95% CI: −406.11, −113.38); sub-group analyses of patients undergoing SSRF or thoracotomy did not detect any significant differences in inpatient opioid consumption between INC and SOC cohorts (**Figure 2D**).

To adjust for LOS differences between cohorts, 14 studies^14–20^^,^^22–26^^,^^28^^,^^33^ reported inpatient opioid use per day (**Table S5**). Nine studies^14^^,^^15^^,^^17–20^^,^^23^^,^^25^^,^^26^ reported a significant reduction in daily opioid consumption, either across the entire postoperative period or on at least one individual postoperative day. Two additional studies^16^^,^^22^ showed reduced daily opioid use with INC, although these studies did not test for statistical significance per postoperative day. A pooled meta-analysis of opioid consumption per postoperative day was not performed due to substantial heterogeneity in reporting across the included studies.

**Figure 3.**
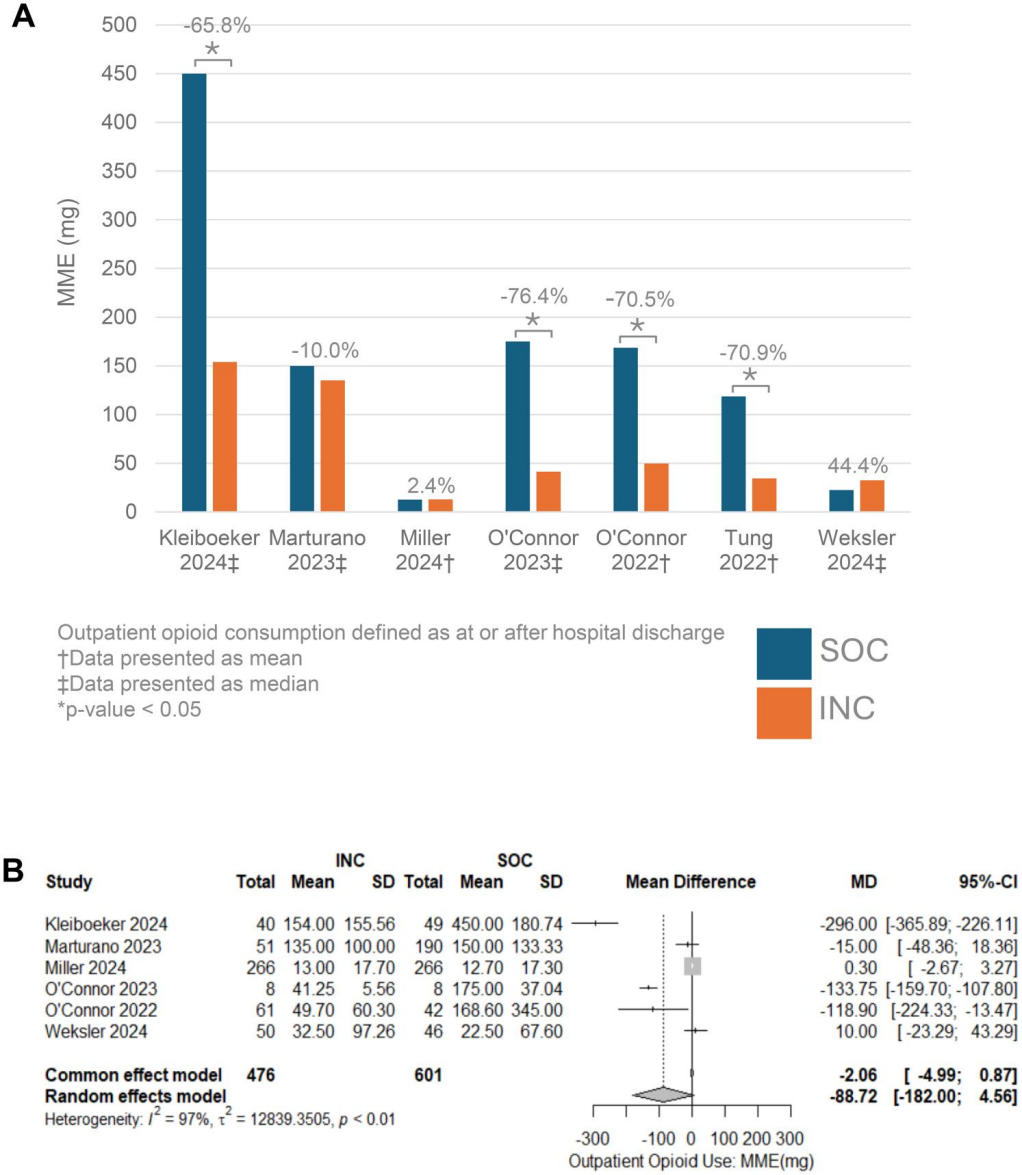
Outpatient opioid consumption. (**A**) Outpatient opioid consumption for INC and SOC cohorts. Percent change in outpatient opioid consumption with INC is reported above each cohort comparison (*P-value < .05). (**B**) Forest plot of outpatient opioid consumption. INC: intercostal nerve cryoablation; MD: mean difference; MME: morphine milligram equivalents; SOC: standard of care

Seven studies^15^^,^^16^^,^^21^^,^^31–33^^,^^35^ reported outpatient opioid consumption. Four studies showed that the INC cohort consumed significantly less opioids compared to the SOC cohort at discharge.^16^^,^^21^^,^^31^^,^^32^ The % reduction in outpatient opioid consumption with INC across studies ranged from 10% to 76.4% (**Figure 3A**).^15^^,^^16^^,^^21^^,^^31^^,^^32^ Two studies reported numerical but non-significant increases in outpatient opioid consumption with INC compared to SOC by 2.4% to 44.4% (**Figure 3A**).^33^^,^^35^ Meta-analysis showed a non-statistically significant reduction in outpatient opioid consumption by 88.72 MME (95% CI: −182.00, 4.56) with INC (**Figure 3B**). The corresponding funnel plot did not suggest evidence of asymmetry, and Egger’s regression test was non-significant (*z* = 1.41; *P* = .16), indicating no evidence of small-study effects or publication bias (**Figure S2**). Sensitivity analysis excluding a database study^35^ revealed significant reductions in outpatient opioid consumption with INC by 108 MME (95% CI: −214.64, −1.35) (**Figure S3**). Sub-group analyses were not performed due to few studies in each sub-group reporting outpatient opioid consumption.

Seven studies reported the percentage of patients discharged with an opioid prescription or with documented opioid use at follow-up. Overall, most studies^16^^,^^17^^,^^19^^,^^22–24^ reported that fewer INC-treated patients required an opioid prescription at discharge and/or outpatient opioids compared to SOC-treated patients, with this difference reaching statistical significance in two studies^17^^,^^19^ (**Table 2**).

**Table 2.** Discharge and Outpatient Opioid Prescriptions and Use

Study	Time point		Patients with opioid prescription, *n* (%)		
			SOC	INC	*P*-value
Choi et al 2021 *J Trauma Acute Care Surg*	2 weeks post-discharge		0 (0%)	3 (27%)	.51
O’Connor et al 2023 *Injury*	Discharge		7 (87.5%)^a^	5 (62.5%)^a^	.56
	Opioid prescription refill 30 days after discharge		1 (13%)	1 (13%)	>.99
Chen et al 2023 *J Surg Res*	Discharge		23 (59.0%)	3 (21.4%)	.02*
McElhinney et al 2024 *Pediatr Blood Cancer*	Discharge		12 (80%)	7 (30.4%)	<.01*
Isaza et al 2023 *Pain Ther*	Outpatient opioid use (follow-up unspecified)		8 (18.6%)	2 (6.9%)	.175
Koons et al 2023 *JTCVS Open*	3-month opioid use		20 (38%)	13 (29%)	NS
	6-month opioid use		14 (27%)	8 (18%)	NS
	12-month opioid use		4 (8%)	6 (14%)	NS
Pourak et al 2024 *Cardiovasc Thorac Surg*	Discharge	TTHHR	41 (95.3%)	28 (84.8%)	.12
		LR	17 (89.5%)	35 (89.7%)	.7
		DLT	29 (90.6%)	16 (80.0%)	.25

a Data reported for opioid naïve patients only.

* *P-*value < .05 for statistical comparison between cohorts; *P-*value reported based on statistical methods in referenced article.

Abbreviations: DLT = double lung transplant; INC = intercostal nerve cryoablation; LR = lung resection; NS = non-significant; SOC = standard of care; TTHHR = transthoracic hiatal hernia repair.

##### Hospital length of stay

**Figure 4.**
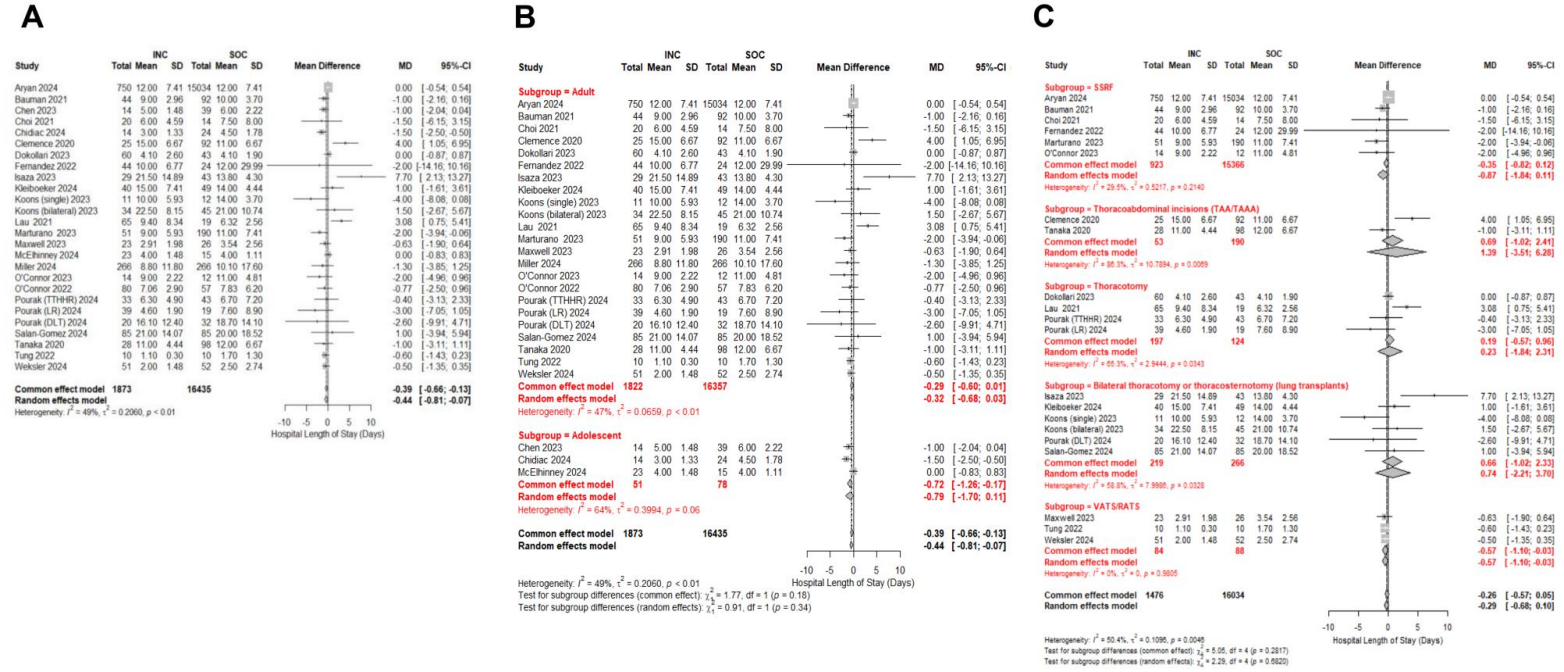
Hospital length of stay. (**A**) Forest plot of hospital LOS. (**B**) Sub-group analyses by patient population age groups. (**C**) Sub-group analyses by surgical approach. Meta-analyses for each individual sub-group are presented in red. The overall pooled results for the common effect and random effects models for all studies are presented in black at the bottom of the panel. DLT: double lung transplant; INC: intercostal nerve cryoablation; LOS: length of stay; LR: lung resection; MD: mean difference; RATS: robotic-assisted thoracic surgery; SOC: standard of care; TAA: thoracic aortic aneurysm; TAAA: thoraco-abdominal aortic aneurysm; TTHHR: transthoracic hiatal hernia repair; VATS: video-assisted thoracic surgery

Twenty-three studies reported on postoperative hospital LOS. The mean LOS for the INC cohort ranged from 1.1 to 22.5 days versus 1.7 to 21 days for those in the SOC cohort. Three studies^12^^,^^15^^,^^18^ reported significantly shorter hospital stay with INC, 2 studies^22^^,^^25^ reported significantly longer hospital stay with INC, and the remaining 18 studies reported no significant difference in hospital LOS with INC compared to SOC. Meta-analysis demonstrated that LOS was significantly shorter with INC by 0.44 (95% CI: −0.81, −0.07; **Figure 4A**). The corresponding funnel plot did not suggest evidence of asymmetry, and Egger’s regression test was non-significant (*z* = 0.59; *P* = .56), indicating no evidence of small-study effects or publication bias (**Figure S4**). Removal of database studies^29^^,^^35^ did not appreciably change the overall LOS difference between INC and SOC in institutional studies (**Figure S5**). When pooled by patient age (adult vs paediatric/adolescent), meta-analysis revealed non-significant changes in hospital LOS with INC in both age populations (0.32-day reduction and 0.79-day reduction, respectively; **Figure 4B**).

Sub-group analyses were carried out to explore the effect of INC on hospital LOS by various surgical approaches. Studies that reported hospital LOS were categorized into the following subgroups based on surgical approach: thoracotomy (for various indications), SSRF, thoracotomy/thoraco-abdominal incisions for thoracic aortic aneurysm (TAA) or thoraco-abdominal aortic aneurysm (TAAA) repair, bilateral thoracotomy or thoracosternotomy (lung transplants), or video- or robotic-assisted thoracic surgery (VATS/RATS). Studies of paediatric/adolescent patient populations were excluded from the surgical approach sub-group analysis.^17–19^ Sub-group analyses based on index procedure revealed no significant difference in hospital LOS with INC in patients who underwent SSRF, thoracotomy, thoracotomy or thoraco-abdominal incisions for TAA or TAAA repair, or lung transplants via bilateral thoracotomy or thoracosternotomy (**Figure 4C**). However, INC was associated with a significant reduction in hospital LOS of 0.57 days (95% CI: −1.10, −0.03) for patients undergoing VATS/RATS (**Figure 4C**).

Six studies reported ICU LOS.^14^^,^^15^^,^^25^^,^^28^^,^^29^^,^^34^ Average ICU LOS ranged from 3 to 9 days for the SOC cohort and 2 to 12 days for the INC cohort. Individually, 3 studies reported a significantly shorter ICU LOS with INC compared to SOC during SSRF,^14^^,^^15^^,^^29^ while the remaining studies reported similar ICU LOS between cohorts.^25^^,^^28^^,^^34^ Meta-analysis demonstrated similar ICU LOS between INC and SOC cohorts (0.12 days; 95% CI: −1.55, 1.79; **Figure S6**).

##### Qualitative analysis

###### Hospital costs/charges

Three studies reported hospital costs/charges, finding no significant difference in total hospital costs/charges between INC and SOC cohorts^12^^,^^16^^,^^35^ (**Table S6**). However, Bauman et al reported higher hospital charges the day of SSRF with INC compared to SOC, while postoperative hospital charges were significantly lower with INC compared to SOC.^12^

###### Pulmonary function

Postoperative pulmonary function was assessed in 6 studies^16^^,^^21^^,^^23^^,^^28^^,^^33^^,^^34^ (**Table S7**). Significant improvements in postoperative pulmonary function were observed in patients treated with INC on the first and second postoperative days following minimally invasive thoracic surgery for lung procedures,^33^ the second postoperative day following minimally invasive heart valve surgery,^28^ and at 6 and 12 months post-lung transplant.^23^^,^^34^

###### Complications

Reported postoperative complications varied by procedure, and not all studies tested for statistical significance between cohorts. Complication rates are presented in **Table S8**. Five studies reported overall complication rates for INC and SOC cohorts,^15^^,^^20^^,^^21^^,^^29^^,^^31^ with 3 finding no significant differences^15^^,^^20^^,^^21^ and 1 reporting significantly lower rates of overall in-hospital complications with INC.^29^ The rates of somnolence,^30^ intestinal disturbance,^30^ pneumonia,^29^^,^^30^ atelectasis,^30^ and pulmonary embolism^29^ were significantly lower with INC compared to SOC, as reported by at least 1 study each. Two studies reported a significant difference in the rate of tracheostomy with conflicting results.^14^^,^^22^ Two studies reported overall rates of pulmonary complications, finding no significant difference between INC and SOC cohorts.^15^^,^^33^ Additionally, there were no significant differences in the rates of 30-day readmission,^15^^,^^16^^,^^18^^,^^21^^,^^31^^,^^35^ reoperation,^15^^,^^19^^,^^20^^,^^25^^,^^27^^,^^29^^,^^31^^,^^34^ or ED visits^17^^,^^19^^,^^35^ between INC and SOC cohorts.

## DISCUSSION

This systematic review and meta-analysis identified 24 studies that compared adjunctive INC to SOC without INC during non-pectus repair thoracic and cardiac surgeries. Cryoablation was associated with a significant reduction in inpatient opioid consumption in adult patients. Overall complication rates were similar or lower compared to other SOC pain management approaches, suggesting INC did not add significant risks.

The benefits of INC in pectus excavatum repair, for which manageable pain is a driver of hospital LOS, have been well documented and include reduced hospital LOS and opioid consumption compared to SOC in adolescents.^8–10^ In contrast, this study included diverse cardiac and thoracic procedures, many of which were complex and invasive. This patient population had more comorbidities and conditions such as traumatic injuries or cancer, which may require repeated exposure to opioids.

### Opioid consumption

The most robust difference between INC and SOC in this patient population was found in opioid consumption. Meta-analysis showed that INC significantly reduced inpatient opioid consumption by 117 MME in the overall pooled analysis, with adult patients experiencing a statistically significant reduction by 102 MME. Outpatient opioid consumption was significantly reduced by 108 MME with INC, when evaluated by sensitivity analysis excluding database studies. Although some studies did not adjust for LOS differences between cohorts, several still found significantly lower opioid usage per postoperative day with INC compared to SOC (**Table S5**).^15^^,^^17–20^^,^^23–26^^,^^28^

Patients undergoing bilateral thoracotomy or thoracosternotomy for lung transplants experienced significant reductions in inpatient opioid consumption with INC by approximately 260 MME—a finding consistent with results from a recent meta-analysis comparing cryoanalgesia to SOC in lung transplant recipients.^36^ Lung transplantation is associated with a substantial postoperative pain burden due to extensive thoracic dissection, rib spreading, and prolonged operative times, creating a wider therapeutic window for analgesic interventions that reduce nociceptive input.^37^ Moreover, because lung transplant recipients are particularly vulnerable to opioid-related adverse effects such as respiratory depression, clinicians often employ more conservative opioid-prescribing practices when effective regional or adjunctive analgesia is available, which may contribute to the pronounced opioid-sparing effect in this group.^38^ While the sub-group analyses were exploratory, the pronounced reduction in opioid use among lung transplant recipients highlights the need for future procedure-specific trials to determine whether certain thoracic populations may derive differential benefit.

Minimizing postoperative opioid consumption is crucial to mitigate the development of adverse side effects and chronic opioid use. An analysis of the Society of Thoracic Surgeons (STS) Adult Cardiac Surgery Database showed that 12.5% of cardiac patients and up to 15.7% of lung surgery patients become newly persistent opioid users.^39^ These data suggest that INC may be an effective strategy to reduce opioid use, concordant with the Enhanced Recovery After Surgery recommendation for opioid-sparing pain management strategies.^40^

### Hospital length of stay

Intercostal nerve cryoablation significantly reduced hospital LOS by approximately half a day overall; however, when stratified by age, LOS remained reduced with INC but did not achieve statistical significance within either adult or paediatric/adolescent subgroups. The reductions in LOS observed herein are less striking than what has been observed in pectus repair patients, where INC resulted in significant reductions in hospital LOS ranging from 1.77 to 2.91 days compared to SOC analgesia.^8^^,^^10^ In the cardiac and thoracic procedures in the current study, hospital LOS may have been strongly influenced by characteristics of this patient population (ie, comorbidities) and index procedure (ie, procedure type, surgical approach, postoperative recovery, etc). Additionally, several nonclinical factors influence hospital LOS such as patient decision-making, discharge destination, and insurance status, suggesting that hospital LOS may be better considered as a process as opposed to an outcome measure.^41^

### Hospital costs/charges

There is concern that INC results in higher healthcare costs due to increased operative time and the additional cost of the cryoablation device. Although hospital costs were only reported by three studies,^12^^,^^16^^,^^35^ INC was cost-neutral in both studies of INC use during SSRF^12^^,^^16^ and in a study of INC use during lobectomy.^35^ However, further research is warranted to evaluate the impact of cryoablation on healthcare costs in other thoracic and cardiac procedures given the limited data on cost-effectiveness in the literature.

### Pulmonary complications and function

Thoracic and cardiac surgical procedures alter respiratory physiology and increase the risk of pulmonary complications (eg, pneumonia, atelectasis). Adequate pain control is necessary to improve respiratory function to help prevent many of these pulmonary-related complications.^42^^,^^43^ In this study, INC was associated with improved respiratory function in half^23^^,^^28^^,^^34^ of the studies that carried out postoperative pulmonary function tests^16^^,^^21^^,^^23^^,^^28^^,^^33^^,^^34^ and was not associated with increased pulmonary complications. While the mechanism for improved pulmonary function with INC is unclear, Salan-Gomez et al suggests that it may be due to improved chest wall mechanics allowing for increased respiratory efforts.^34^ However, tests of pulmonary function are challenging to perform due to physical and physiological constraints such as getting into the optimal position, prolonged oral intubation, and hypoxia, which may result in confounding variables and/or missing values for pulmonary function measurements for many patients.^28^ Future research is warranted to understand the mechanism by which INC improves pulmonary function.

### Neuropathic pain

Neuropathic pain is common after thoracotomy, and a historical concern with INC is the development of postoperative neuralgia. Although sparsely reported, none of the publications identified in this systematic review documented significantly increased rates of neuropathic symptoms in patients treated with INC. Included studies generally reported low rates of persistent neuropathic symptoms and frequent resolution over time; however, long-term follow-up was limited and reporting was inconsistent. A single study used the Leeds Assessment of Neuropathic Symptoms and Signs (LANSS) 2 weeks post-discharge, reporting higher scores and more patients with scores ≥12 with INC, the latter indicative of neuropathic pain.^33^ Furthermore, future studies should incorporate standardized, long-term assessments of neuropathic symptoms to more definitively characterize the chronic pain risks associated with INC. The details of neuropathic symptoms and resolution reported by studies are presented in **Table S9**.

### Overall complication rates, readmission, and reoperation

The safety profile of INC is highlighted by comparable rates of overall complications, readmission, and reoperation between SOC and INC cohorts. Furthermore, in some reports, INC was associated with significantly lower rates of pulmonary and opioid-related complications. Thus, enhanced recovery may be possible with INC as shown by the lower incidence of certain complications. Importantly, none of the studies reported INC device-related complications, underscoring the safety of currently available INC devices in this patient population.

### Limitations

This study has several limitations. The protocol for this systematic review and meta-analysis was not registered in a public database; however, it was developed prior to the study initiation and adhered to with only minor modifications. Study screening and duplicate removal were performed manually by 2 independent reviewers without the use of dedicated screening software, which may limit the reproducibility of the study selection process. Included studies were primarily single-center, retrospective, observational studies which may limit the generalizability of the findings and introduce selection bias and confounding variables. Only three prospective, RCTs (2 single-center^30^^,^^33^ and 1 multicentre^28^) were identified.

While the findings suggest that improved outcomes are associated with INC, these results should be interpreted cautiously due to substantial heterogeneity among included studies in patient populations, surgical techniques, and the definitions of SOC analgesia. The SOC was not a uniform comparator and encompassed diverse strategies—including thoracic epidural, paravertebral and peripheral nerve blocks, and multimodal regimens. Although a more granular sub-group or sensitivity analysis based on SOC analgesia type would have been desirable, such analyses were not feasible due to inconsistent and often limited reporting of SOC components across studies, many of which were retrospective and lacked a protocol-defined analgesic regimen. As a result, meaningful stratification by SOC type could not be performed without substantial risk of misclassification. Regardless, this heterogeneity reflects real-world practice and may enhance the generalizability.

Surgical procedures also varied in magnitude, influencing outcomes through differences in invasiveness and tissue trauma. Sub-group analyses by surgical approach were performed using only studies with homogenous cohorts with a specific surgical approach, with the aim to explore the potential differential impact of surgical approach with as much control as feasible given constraints in data reporting. Similarly, age-based sub-group analyses were conducted, but the predominance of adult data limits applicability to paediatric/adolescent populations.

Moreover, variability in reporting methods—such as units used for opioid consumption and the statistical approaches applied—required certain assumptions to be made for evaluation of synthesized data. For inpatient opioid consumption, the pooled estimate should be interpreted cautiously because the corresponding funnel plot demonstrated significant asymmetry by Egger’s test, suggesting potential publication bias or small-study effects, which may partly reflect heterogeneity in opioid use-measurement methods, analgesic protocols, or patient populations rather than selective publication alone.

Importantly, random effects modelling was employed for meta-analyses to account for the substantial heterogeneity across studies. Random effects models assume that true effect sizes may vary across studies due to differences in populations, interventions, and methodologies—thereby enhancing the generalizability of the findings across diverse clinical settings. Random effects modelling also yields more conservative estimates by incorporating between-study variability, reducing the risk of overestimating the precision or magnitude of the observed effects. Regardless, the significant heterogeneity detected across included studies underscores the need for future studies designed with standardized comparators, outcome measures, and age-specific analyses to strengthen evidence for INC and its potential benefits.

## CONCLUSION

The results herein qualitatively and quantitatively aggregate published data on INC compared to SOC in cardiac and thoracic procedures including lung transplants, lobectomies, resections, decortications, minimally invasive structural heart surgeries, TTHHR, aortic aneurysm repair, paediatric/adolescent cancer surgery, and SSRF. Across these diverse procedures, the most significant and clinically meaningful finding was that adult patients receiving INC had significantly lower inpatient opioid use than those receiving SOC. Randomized controlled trials in more homogenous patient groups may help to identify additional INC benefits in specific sub-populations.

## Supplementary Material

ivag143_Supplementary_Data

## Data Availability

The protocol and datasets generated during and/or analysed during the current study are available from the corresponding author on reasonable request.

## ABBREVIATIONS

DLT: double lung transplant
ED: emergency department
ICU: intensive care unit
INC: intercostal nerve cryoablation
IQR: interquartile range
LOS: length of stay
LR: lung resection
MME: morphine milligram equivalents
NR: not reported
NS: non-significant
PRISMA: Preferred Reporting Items for Systematic reviews and Meta-Analyses
RATS: robotic-assisted thoracic surgery
RCT: randomized controlled trials
SOC: standard of care
SSRF: surgical stabilization of rib fractures
TAA: thoracic aortic aneurysm
TAAA: thoraco-abdominal aortic aneurysm
TTHHR: transthoracic hiatal hernia repair
VATS: video-assisted thoracic surgery
