# Supplementary material for: Intercostal Nerve Cryoablation Therapy in Thoracic and Cardiac Surgery for Postoperative Pain Management: A Systematic Review and Meta-Analysis
Source: Interdiscip Cardiovasc Thorac Surg. 2026 May 23;41(6):ivag143. doi: 10.1093/icvts/ivag143 (PMC13251889; doi:10.1093/icvts/ivag143)
Supplement: ivag143_Supplementary_Data [file ivag143_supplementary_data.zip › Supplementary_Data.docx]

Intercostal Nerve Cryoablation Therapy in Thoracic and Cardiac Surgery for Postoperative Pain Management: A Systematic Review and Meta-Analysis

Christopher W. Towe, MD^1^, Zachary M. Bauman, DO, MHA, FACOS, FACS^2^, Lizabeth A. O’Connor, DMSc, PA-C, MPH^3^, Curtis C. Quinn, MD^3^, Marc P. Pelletier, MD, MSc, FRCSC^4^, Alyssa K. Hahn, PhD^5^, Nfii Ndikintum, PhD^5^, Madelyn M. Dua, PhD^5^, Edward Cantu, MD, MSCE^6^

^1^University Hospitals Cleveland Medical Center, Department of Surgery, Division of Thoracic and Esophageal Surgery, Cleveland, OH, USA

^2^University of Nebraska Medical Center, Department of Surgery, Division of Acute Care Surgery, Omaha, NE, USA

^3^Elliot Health System, Division of Thoracic Surgery, Manchester, MA, USA

^4^Yale New Haven Hospital, Division of Cardiac Surgery, New Haven, CT, UAS

^5^AtriCure, Inc., Mason, OH, USA

^6^Hospital of the University of Pennsylvania, Department of Surgery, Division of Cardiovascular Surgery, Philadelphia, PA, USA

Corresponding Author:

Christopher W. Towe, MD

University Hospitals Cleveland Medical Center, Division of Thoracic and Esophageal Surgery, Department of Surgery

11100 Euclid Avenue, Cleveland, OH 44106, USA

Email: Christopher.Towe@UHhospitals.org

**Supplementary Material**

**Supplemental Tables**

**Supplemental Table 1.** Full search strings

| **Search No.** | **Search Term/String** |
| --- | --- |
| 1 | (“cryoablation” OR “cryoanalgesia” OR “cryoneurolysis” OR “cryo”) AND ("intercostal nerve" OR “intercostal” OR "nerve block") AND ("2014/11/25"[Date - Publication] : "3000"[Date - Publication]) NOT (“Nuss” or “Ravitch” OR “pectus”) NOT (“percutaneous”) |
| 2 | ("cryoablation" OR "cryoneurolysis" OR "cryoanalgesia" OR "cryo") AND ("pain" OR "opioid”) AND (“surgery”) AND ("2014/11/25"[Date - Publication] : "3000"[Date - Publication] NOT (“percutaneous”) NOT (“cryosurgery” OR “tumor”) NOT (“Nuss” OR “Ravitch” OR “pectus”) |
| 3 | ("Intercostal" OR "peripheral") AND ("nerve block" OR "cryoablation" OR "cryoneurolysis" OR "cryoanalgesia" OR "cryo") AND ("AtriCure" OR "CryoNB" OR "cryoICE" OR "cryoprobe" OR "cryoSPHERE") AND ("2014/11/25"[Date - Publication] : "3000"[Date - Publication]) NOT (“percutaneous”) NOT (“Nuss” OR “Ravitch” OR “pectus”) NOT (“cryosurgery” OR “tumor”) |
| 4 | (“cardiac” OR “thoracic” OR “thorax” OR "heart" OR "chest" OR "lung" OR “rib”) AND ("intercostal" OR "peripheral") AND ("cryo" OR "cryoablation" OR "cryoanalgesia" OR "cryoneurolysis") AND ("2014/11/25"[Date - Publication] : "3000"[Date - Publication]) NOT (“percutaneous”) NOT (“Nuss” OR “Ravitch” OR “pectus”) NOT (“cryosurgery” OR “tumor”) |

**Supplemental Table 2.** Quality and risk-of-bias assessments: cohort studies (Newcastle Ottawa scale)

|  | Selection (max 4 points) | Comparability (max 2 points) | Outcome (max 3 points) | Total points (max 9 points) |
| --- | --- | --- | --- | --- |
| Aryan 2024 | 3 | 0 | 3 | 6 |
| Bauman 2021 | 3 | 0 | 3 | 6 |
| Choi 2021 | 4 | 1 | 3 | 8 |
| Clemence 2020 | 3 | 1 | 3 | 7 |
| Dokollari 2023 | 4 | 2 | 3 | 9 |
| Fernandez 2022 | 3 | 1 | 3 | 7 |
| Kleiboeker 2024 | 3 | 1 | 3 | 7 |
| Marturano 2023 | 4 | 1 | 3 | 8 |
| Miller 2024 | 3 | 0 | 3 | 6 |
| O’Connor 2023 | 4 | 1 | 3 | 8 |
| Chen 2023 | 4 | 1 | 3 | 8 |
| McElhinney 2024 | 4 | 1 | 3 | 8 |
| Chidiac 2024 | 3 | 1 | 3 | 7 |
| Isaza 2023 | 4 | 1 | 3 | 8 |
| Koons 2023 | 3 | 2 | 3 | 8 |
| Maxwell 2023 | 4 | 1 | 3 | 8 |
| O’Connor 2022 | 4 | 1 | 3 | 8 |
| Pourak 2024 | 4 | 1 | 3 | 8 |
| Salan-Gomez 2024 | 3 | 1 | 3 | 7 |
| Tanaka 2020 | 4 | 1 | 3 | 8 |
| Tung 2022 | 4 | 2 | 3 | 9 |

**Supplemental Table 3.** Quality and risk-of-bias assessments: randomized controlled trials (RoB 2)

| **Risk of Bias Domain** | **Lau 2021** | **Weksler 2024** | **Ba 2015** |
| --- | --- | --- | --- |
| Randomization process | Low | Low | Low |
| Assignment to intervention | Low | Low | Low |
| Missing outcome data | Low | Low | Low |
| Measurement of outcome | Low | Low | Low |
| Selection of the reported result | Low | Low | Low |
| Overall risk of bias | Low | Low | Low |

**Supplemental Table 4.** Intervention characteristics

| **Study** | **Treatment Arm** | **Cryoablation Device** | **Cryoablation Temperature** (◦C) | **Freeze Duration Per Nerve** | **Number of Nerves Ablated** | **Comparison Cohort Pain Management Approach** |
| --- | --- | --- | --- | --- | --- | --- |
| Bauman et al. 2021 *Injury* | INC | AtriCure cryoICE (unspecified model) | -60 | 2 min | Intercostal nerves of all fractured ribs underwent cryoablation staying within T3-T9 | NA |
|  | SOC | NA | NA | NA | NA | Multimodal pain regimen without INC |
| Choi et al. 2021 *J Trauma Acute Care Surg* | INC | AtriCure cryoICE cryoSPHERE | -60 to -80 | 2 min | 6 (6-7) | NA |
|  | SOC | NA | NA | NA | NA | Multimodal analgesia without INC |
| Fernandez et al. 2022 *J Trauma Acute Care Surg* | INC | AtriCure cryoICE cryoSPHERE | -60 to -65 | 2 min | 5 (4-6) | NA |
|  | SOC | NA | NA | NA | NA | Standard multimodal analgesia without INC |
| Marturano et al. 2023 *Injury* | INC | AtriCure cryoICE (unspecified model) | NR | 2 min | T3-T9; 6 (5-6) | NA |
|  | SOC | NA | NA | NA | NA | Standard multimodal pain regimen without INC |
| O'Connor et al. 2023 *Injury* | INC | AtriCure cryoICE cryoSPHERE | -50 to -70 | 2 min | at level of each rib fracture, one level above the highest fracture and one level below the most inferior fracture; taking care to avoid freezing the 3rd intercostal space or below the 9th | NA |
|  | SOC | NA | NA | NA | NA | Elastomeric infusion pump |
| Aryan 2024 *J Surg Res* | INC | NR | -40 to -70 | 2 min | NR | NA |
|  | SOC | NA | NA | NA | NA | No INC |
| Chen et al. 2023 *J Surg Res* | INC | AtriCure cryoICE cryoSPHERE | NR | NR | 5; at the intercostal level of the thoracotomy as well as two spaces above and two spaces below | NA |
|  | SOC | NA | NA | NA | NA | Various non-INC approaches; predominantly epidural and patient-controlled analgesia |
| Chidiac et al. 2024 *J Surg Res* | INC | AtriCure cryoICE cryoSPHERE | NR | 2 min | In most cases, cryoablation probe was applied to fifth intercostal space as well as two intercostal spaces above and two below | NA |
|  | SOC | NA | NA | NA | NA | No INC |
| McElhinney et al. 2024 *Pediatr Blood Cancer* | INC | AtriCure cryoICE cryoSPHERE | NR | 60-120 sec (median 90 sec) | range: 2-7; median of 5 intercostal nerves | NA |
|  | SOC | NA | NA | NA | NA | Routine, multimodal pain management (67% with regional block, n=10) |
| Ba et al. 2015 *Surg Today* | INC | NR | -55 to -65 | 90 sec | 4 intercostal nerves; one at the level of the incision, one each at the levels above and below the incision, and one at the level of the drainage tube that was placed at the end of the operation | NA |
|  | SOC | NA | NA | NA | NA | Intravenous parecoxib |
| Maxwell et al. 2023 *Innovations* | INC | AtriCure cryoICE (unspecified model) | NR | 2 min | 5 intercostal nerves; T3-T7 | NA |
|  | SOC | NA | NA | NA | NA | Routine pain management program without INC |
| Miller et al. 2024 *Pain Ther* | INC | AtriCure cryoICE (unspecified model) | -50 to -70 | 2 min | Typically five levels | NA |
|  | SOC | NA | NA | NA | NA | Non-standardized SOC included epidural, regional blocks, opioids, non-opioids, etc. |
| O'Connor et al. 2022 *J Surg Res* | INC | AtriCure cryoICE cryoSPHERE | -50 to -70 | NR | Average 5 ablations between T3-T9 (typically two intercostal spaces above, two below, and one at the incision level) | NA |
|  | SOC | NA | NA | NA | NA | Standard analgesic treatment including opioid and non-opioid medication (5 patients receiving epidurals) |
| Tung et al. 2022 *J Robot Surg* | INC | Frigitronics CE-82 cryosurgical system and probe | -80 | 2 min | T7-T10 | NA |
|  | SOC | NA | NA | NA | NA | Standard multimodal pain regimen that included preoperative administration of acetaminophen and gabapentin and intraoperative fentanyl and paravertebral nerve blocks |
| Isaza et al. 2023 *Pain Ther* | INC | AtriCure cryoICE cryoSPHERE | -60 | NR | T3-T7 | NA |
|  | SOC | NA | NA | NA | NA | Thoracic epidural analgesia |
| Kleiboeker et al. 2024 *JHLT Open* | INC | AtriCure cryoICE (unspecified model) | -60 to -80 | 2 min | T3-T7 | NA |
|  | SOC | NA | NA | NA | NA | No INC |
| Koons et al. 2023 *JTCVS Open* | INC | AtriCure cryoICE (unspecified model) | -50 to -70 | 2 min | 4; at intercostal space of entry, 1 space above and 2 spaces below | NA |
|  | SOC | NA | NA | NA | NA | Standard pain management without INC |
| Pourak et al. 2024 *Cardiovasc Thorac Surg* | INC | AtriCure cryoICE cryoSPHERE | -70 | 2 min | 5 intercostal spaces; 2 spaces above thoracotomy, the thoracotomy space, and 2 spaces below | NA |
|  | SOC | NA | NA | NA | NA | Preoperative thoracic epidurals and patient-controlled analgesia |
| Salan-Gomez et al. 2024 *JTCVS Open* | INC | AtriCure cryoICE cryoSPHERE | -70 | 2 min | T3-T7 | NA |
|  | SOC | NA | NA | NA | NA | Multimodal pain regimen without INC; relied primarily on opioid use |
| Weksler et al. 2024 *JTCVS* | INC | AtriCure cryoICE cryoSPHERE | -80 | 2 min | 5-6 intercostal nerves; the nerves above and below the intercostal space where the ports were located were always included in the 5-6 nerves ablated | NA |
|  | SOC | NA | NA | NA | NA | Internal intercostal block from the 2nd intercostal nerve to the 10th intercostal nerve using a mixture of 1:1 bupivacaine (0.5%) with epinephrine (1:200,000) and lidocaine (2%) |
| Clemence et al. 2020 *Semin Thorac Cardiovasc Surg* | INC | AtriCure cryoICE (unspecified model) | -60 | 2 min | T3-T9 if 2 thoracotomies in the 4th and 7th/8th intercostal spaces were used; seventh or eighth space was used; if only 1 thoracotomy was used, 2 intercostal nerves above and below (T3-T7 if thoracotomy in 5th intercostal space; T5 or T6-9 if thoracotomy in 7th/8th intercostal space) | NA |
|  | SOC | NA | NA | NA | NA | Standard pain management (narcotic and non-narcotic medication) |
| Tanaka et al. 2020 *Ann Thorac Surg* | INC | AtriCure cryoICE CRYO2 | -60 | 2 min | T4-T8 (TAA repair) or T4-T10 (TAAA repair) | NA |
|  | SOC | NA | NA | NA | NA | Peripheral nerve block with standard postoperative pain regimen |
| Dokollari et al. 2023 *Rev Cardiovasc Med* | INC | AtriCure cryoICE (unspecified model) | -65 | 2 min | 2 levels above incision, at the level of the incision, and 2 levels below the lowest incision | NA |
|  | SOC | NA | NA | NA | NA | Standardized multimodal analgesia without INC |
| Lau et al. 2021 *Pain Ther* | INC | AtriCure cryoICE CRYO2 | -50 to -70 | 2 min | at level of intercostal incision and 2 levels above and 2 levels below | NA |
|  | SOC | NA | NA | NA | NA | Standard of care postoperative pain management |
| INC: intercostal nerve cryoablation; NR: not reported; SOC: standard of care; TAA: thoracic aortic aneurysm; TAAA: thoracoabdominal aortic aneurysm | | | | | | |
| Number of nerves ablated data reported as median (IQR), unless otherwise indicated | | | | | | |

**Supplemental Table 5.** Daily opioid consumption

| **Study** | **Description** |  | **SOC** | **INC** | | **p-value** |
| --- | --- | --- | --- | --- | --- | --- |
| Chen et al. 2023 *J Surg Res* | Daily opioid consumption, median, MME/kg^†^ | POD3 | 0.028 | 0.0 | | <0.05* |
|  |  | POD4 | 0.33 | 0.0 | | <0.05* |
|  |  | POD5 | 0.36 | 0.0 | | <0.05* |
| Chidiac et al. 2024 *J Surg Res* | Opioid use per postoperative day, median, OME/kg | POD0 | 1.2 | 0.5 | | <0.05* |
|  |  | POD1 | 2.8 | 0.2 | | <0.05* |
|  |  | POD2 | 2.5 | 0.5 | | <0.05* |
|  |  | POD3 | 2.4 | 0.3 | | <0.05* |
|  |  | POD4 | 1.6 | 0.8 | | NS |
|  |  | POD5 | 2.3 | 0.1 | | <0.05* |
|  |  | POD6 | 3.8 | 0.0 | | <0.05* |
| Clemence et al. 2020 *Semin Thoracic Surg* | Total narcotic usage per patient per post-extubation day 1 to day 10 or discharge, mean (standard error), MME (mg) | Day 1 | 42 (3) | 24 (2) | | 0.006* |
|  |  | Day 2 | 52 (4) | 31 (3) | | 0.006* |
|  |  | Day 3 | 54 (5) | 33 (3) | | 0.01* |
|  |  | Day 4 | 59 (4) | 25 (3) | | 0.0002* |
|  |  | Day 5 | 54 (3) | 26 (3) | | 0.0005* |
|  |  | Day 6 | 52 (5) | 28 (3) | | 0.004* |
|  |  | Day 7 | 54 (6) | 24 (3) | | 0.001* |
|  |  | Day 8 | 60 (8) | 24 (3) | | 0.002* |
|  |  | Day 9 | 67 (11) | 16 (2) | | 0.0004* |
|  |  | Day 10 | 68 (10) | 13 (2) | | 0.0005* |
| Fernandez et al. 2022 *J Trauma Acute Care Surg* | Daily MME (mg), median (95% CI) |  | 62.2 (37.9-102.0) | 28.5 (19.8-41.1) | | 0.014* |
| Koons et al. 2023 *JTCVS Open* | Oral opioid use per day, MME (mg) | POD3 | 20.5 | | 12.3 | <0.05* |
|  |  | POD7 | 39.9 | | 23.4 | <0.05* |
|  |  | POD14 | 41.6 | | 22.6 | <0.05* |
|  |  | POD21 | 28.2 | | 15.9 | NS |
| Lau et al. 2021 *Pain Ther* | Opioid consumption per day for entire postoperative stay, mean (SD), MME (mg) |  | 38.31 (31.05) | 33.43 (29.77) | | >0.05 |
| Marturano et al. 2023 *Injury* | Daily MME (mg), median (IQR) |  | 32.8 (15.2, 57.6) | 16.3 (8, 36.7) | | 0.01* |
| Maxwell et al. 2023 *Innovations* | Morphine equivalent dosages per postoperative day | POD1 | 47.24 | 5 | | <0.001* |
|  |  | POD2 | 25.04 | 10.93 | | 0.003* |
|  |  | POD3 | 21.71 | 8.13 | | 0.009* |
|  |  | POD4 | 19.17 | 7.08 | | 0.011* |
| McElhinney et al. 2024 *Pediatr Blood Cancer* | Opioid utilization per day, OME (mg)/kg/day, median (IQR) |  | 1.72 (1.19-2.47) | 0.64 (0.25-1.03) | | <0.001* |
| Pourak et al. 2024 *Cardiovasc Thorac Surg* | Average oral narcotic usage on the day prior to discharge, MME (mg), mean (SD) | TTHHR | 18.8 (22.0) | 14.4 (17.5) | | 0.48 |
|  |  | LR | 28.3 (36.2) | 24.8 (30.1) | | 0.96 |
|  |  | DLT | 29.5 (32.1) | 40.1 (48.2) | | 0.81 |
| Tanaka et al. 2020 *Ann Thorac Surg* | Total amount of postoperative opioid use in MME (mg) per day, median (IQR) | POD1 | 72 (36-120) | 57.3 (15-93.5) | | 0.122 |
|  |  | POD2 | 48 (20-96) | 48.1 (21.9-91.7) | | 0.847 |
|  |  | POD3 | 47.5 (20-86.8) | 30 (5-53.1) | | 0.058 |
|  |  | POD4 | 35 (15-60.5) | 15 (5-37) | | 0.006* |
|  |  | POD8 | 32.5 (14-63) | 0 (0-11.3) | | <0.001* |
| Weksler et al. 2024 *JTCVS* | Inpatient opioid use per day, MME (mg), median (IQR) |  | 21.2 (10, 37.1) | 20.8 (9.3, 33) | | 0.702 |
| ^†^Median opioid use values (MME/kg) were not reported for postoperative days in which the difference between groups was not statistically significant (PODs 0, 1, 2, and 6). | | | | | | |
| *p-value<0.05 for statistical comparison between cohorts; p-values reported based on statistical methods in referenced article | | | | | | |
| Only studies that reported exact numerical values for opioid use per day are included in the table. Studies that presented opioid use data only in graphical form were excluded unless exact values were explicitly stated in the text or figure. Opioid use per day was not estimated from figures. Two studies (O’Connor et al. 2023 and Isaza et al.) reported daily opioid use graphically without specific values or statistical comparisons per postoperative day reported in text; however, both studies showed reduced daily opioid use with INC. | | | | | | |
| DLT: double lung transplant; INC: intercostal nerve cryoablation; IQR: interquartile range; LR: lung resection; MME: morphine milligram equivalent; NS: non-significant; OME: oral morphine equivalent; POD: postoperative day; SD: standard deviation; SOC: standard of care; TTHHR: transthoracic hiatal hernia repair. | | | | | | |

**Supplemental Table 6.** Hospital charges and costs

| **Study** | **Hospital Charge/Cost** | **SOC** ($) | **INC** ($) | **p-value** |
| --- | --- | --- | --- | --- |
| Bauman et al. 2021 *Injury* | Total hospital charges | 143,196 (109,183 - 212,318) | 153,908 (118,820-213,823) | 0.734 |
|  | Hospital charges day of SSRF | 71,143 (52,401-86,611) | 93,932 (79,384-114,292) | <0.001* |
|  | Postoperative hospital charges | 20,269 (10,445-43,430) | 10,556 (4,481-23,026) | 0.001* |
| O'Connor et al. 2023 *Injury* | Total hospital charges (all patients) | 131,498 (73,072) | 90,224 (34,633) | 0.07 |
|  | Total hospital charges (opioid naïve) | 125,161 (69,776) | 91,080 (37,440) | 0.24 |
|  | Total hospital charges (opioid tolerant) | 144,170 (88,868) | 89,083 (33,963) | 0.19 |
| Miller et al. 2024 *Pain Ther* | Index total costs | 43,974 (41,879) | 38,753 (29,513) | 0.10 |
| INC: intercostal nerve cryoablation; SOC: standard of care | | | | |
| Data presented as median (interquartile range) or mean (standard deviation) | | | | |
| *p-value<0.05 for statistical comparison between cohorts; p-values reported based on statistical methods in referenced article | | | | |
| Costs/charges presented in USD as reported and were not adjusted for inflation | | | | |

**Supplemental Table 7.** Pulmonary function

| **Study** | **Time Points** | **Pulmonary Function** | | | |
| --- | --- | --- | --- | --- | --- |
|  |  | **Parameter** | **SOC** | **INC** | **p-value** |
| O'Connor et al. 2023 *Injury* | Baseline and POD1-3 | incentive spirometry effort (mL) | raw values not reported | raw values not reported | 0.71 |
| Koons et al. 2023 *JTCVS Open* | 1 month | percent predicted FEV1 | 67.5 (58-83) | 66 (59-74) | NR |
|  | 3 months | percent predicted FEV1 | 71.5 (60.5-89) | 68 (58-77) | NR |
|  | 6 months | percent predicted FEV1 | 67 (57-79) | 73 (54-87) | NR |
|  | 12 months | percent predicted FEV1 values | 63.5 (57-84) | 83.5 (73-88) | NR |
| O'Connor et al. 2022 *J Surg Res* | POD1 | incentive spirometry effort (mL) | 1141 (634) | 1386.7 (737.9) | 0.052 |
|  | POD2 | incentive spirometry effort (mL) | 1169 (646) | 1381.5 (709.1) | 0.07 |
|  | Day of discharge | incentive spirometry effort (mL) | 1300 (609.4) | 1518.9 (714.5) | 0.06 |
| Salan-Gomez 2024 *JTCVS Open* | 3 months | percent FEV1 | 62%† | 70%† | 0.052 |
|  | 6 months | percent FEV1 | 68% | 78% | 0.007* |
|  | 12 months | percent FEV1 | 69% | 83% | 0.006* |
| Weksler et al. 2024 *JTCVS* | POD1 | incentive spirometry effort (mL) | 1000 (750-1075) | 1250 (1000-2000) | <0.001* |
|  | POD2 | incentive spirometry effort (mL) | 1000 (750-1500) | 1500 (1000-2000) | 0.012* |
|  | POD3 | incentive spirometry effort (mL) | 1000 (750-1712) | 1500 (937-2000) | 0.276 |
|  | Preop to day 1 | incentive spirometry effort (mL) | -875 (-1250, -500) | -750 (-1250, -500) | 0.330 |
|  | Preop to day 2 | incentive spirometry effort (mL) | -500 (-1000, -250) | -500 (-1000, 0) | 0.649 |
|  | Preop to day 3 | incentive spirometry effort (mL) | -500 (-750, 0) | -375 (-1000, 0) | 0.961 |
| Lau et al. 2021 *Pain Ther* | POD2 | FEV1 (L) | 0.93 (0.43) | 1.20 (0.46) | 0.02* |
|  | POD3 | FEV1 (L) | 1.19 (0.52) | 1.25 (0.56) | NS |
|  | POD4 | FEV1 (L) | 1.19 (0.57) | 1.40 (0.58) | NS |
|  | POD5 | FEV1 (L) | 0.96 (0.67) | 1.40 (0.71) | NS |
| FEV1: forced expiratory volume in 1 second; INC: intercostal nerve cryoablation; NR: not reported; NS: non-significant; POD: postoperative day; SOC: standard of care | | | | | |
| *p-value<0.05 for statistical comparison between cohorts; p-values reported based on statistical methods in referenced article | | | | | |
| Data presented as median (IQR) or median (SD), unless otherwise indicated | | | | | |
| †Values were estimated from the graphical representation in original publication | | | | | |

**Supplemental Table 8.** Complications

| **Complication** | **Study** | **INC** | **SOC** | **p-value** |
| --- | --- | --- | --- | --- |
| **Overall Complications, n (%)** | | | | |
|  | Aryan et al. 2024 | 153 (20.4%); in-hospital complications | 3670 (24.4%); in-hospital complications | 0.01 |
|  | Marturano et al. 2023 | 10 (19.6%) | 38 (20%) | 1 |
|  | Maxwell et al. 2023 | 9 (39.1%); any complication | 7 (26.9%); any complication | 0.363 |
|  | O'Connor et al. 2022 | 4 (5.2%); any complication | 6 (10.9%); any complication | 0.31 |
|  | Tung et al. 2022 | 2 (20%)† | 5 (50%)† | NR |
| **Neuropathic Symptoms, n (%)** | | | | |
| Allodynia | Lau et al. 2021 | 1 (1.6%) at 6-months† | 0 (0%)† | NR |
| Neuralgia | O'Connor et al. 2023 | 0 (0%) at 1-yr | 2 (17%) at 1-yr | 0.11 |
|  | Chen et al. 2023 | Two patients reported variable duration of numbness and one cryoablation patient reported burning sensation at the incision site; all patients had resolution of numbness or burning sensation by their 6-mo. outpatient follow-up† | 0 (0%)† | NR |
|  | O'Connor et al. 2022 | 1 (1.3%); uncontrolled pain requiring readmission | 0 (0%); uncontrolled pain requiring readmission | 1 |
| Neuropathic pain | Tung et al. 2022 | 1 (10%)† | 1 (10%)† | NR |
| Paresthesia | Choi et al. 2021 | 1 (6%); 6-month follow-up (mild)† | 0 (0%); 6-month follow-up† | NR |
|  | Fernandez et al. 2022 | 4 (10.5%) short-term follow-up 0-3 months; 0 (0%) long-term follow-up 3-6 months† | 6 (28.6%) short-term follow-up 0-3 months; 3 (50%) long-term follow-up 3-6 months† | NR |
| **Opioid-Related Symptoms, n (%)** | | | | |
| Gastrointestinal disturbances | Ba et al. 2015 | 0 (0%); intestinal disturbance | 7 (7.69%); intestinal disturbance | <0.05* |
|  | Tanaka et al. 2020 | 1 (4%); gastrointestinal complications - ileus and bleeding | 9 (9%); gastrointestinal complications - ileus and bleeding | 0.456 |
| Drowsiness/somnolence | Ba et al. 2015 | 0 (0%) | 4 (4.40%) | <0.05* |
| Delirium/hallucinations | Aryan et al. 2024 | 21 (5.5%) | 204 (5.5%) | 1 |
| **Pulmonary Complications, n (%)** | | | | |
| Pulmonary Complications | Marturano et al. 2023 | 10 (19.6%); multivariable results - adjusted for covariates (age) | 38 (20.0%); multivariable results - adjusted for covariates (age) | 0.693; multivariable analysis results; adjusted for covariates (age) |
|  | Weksler et al. 2024 | 3 (5.9%) | 8 (15.4%) | 0.12 |
| Pneumonia | Aryan et al. 2024 | 16 (2.1%); ventilator-associated pneumonia (hospital acquired infection) | 572 (3.8%); ventilator-associated pneumonia (hospital-acquired infection) | 0.02* |
|  | Choi et al. 2021 | 0% | 2 (14%) | 0.16 |
|  | Fernandez et al. 2022 | 15.9% (95% CI: 8.0-31.8%) | 33.3% (95% CI: 18.7-59.3%) | 0.106 |
|  | Ba et al. 2015 | 2 (2.30%) | 4 (4.40%) | <0.05* |
|  | Miller et al. 2024 | 33 (12.4%) | 20 (7.5%) | 0.08 |
|  | O'Connor et al. 2022 | 0 (0%) | 3 (5.5%) | 0.06 |
|  | Salan-Gomez et al. 2024 | 22 (26%) | 17 (20%) | 0.362 |
|  | Clemence et al. 2020 | 4 (16%) | 9 (9.8%) | 0.47 |
|  | Tanaka et al. 2020 | 1 (4%) | 0 (0%) | 0.228 |
| Tracheostomy | Choi et al. 2021 | 1 (5%) | 1 (7%) | 0.99 |
|  | Fernandez et al. 2022 | 4.6% (95%: CI: 1.1-18.1%) | 25% (95% CI: 12.3-50.6%) | 0.032* |
|  | Marturano et al. 2023 | 3 (5.9%) | 7 (3.7%) | 0.88 |
|  | Isaza et al. 2023 | 12 (41.7%) | 5 (11.6%) | 0.006* |
|  | Salan-Gomez et al. 2024 | 26 (31%) | 26 (31%) | >0.99 |
| Persistent Air Leak | Chidiac et al. 2024 | 2 (14.3%) | 7 (29.3%) | 0.26 |
|  | Maxwell et al. 2023 | 3 (13.0%) | 4 (15.4%) | 0.815 |
|  | Miller et al. 2024 | 19 (7.1%); lung leak | 23 (8.6%); lung leak | 0.63 |
|  | Tung et al. 2022 | 0 (0%)† | 2 (20%)† | NR |
|  | Weksler et al. 2024 | 5 (9.8%); air leak >5 days | 9 (17.3%); air leak >5 days | 0.27 |
| Pneumothorax | Maxwell et al. 2023 | 1 (4.3%) | 0 (0%) | 0.283 |
|  | Miller et al. 2024 | 39 (14.7%) | 30 (11.3%) | 0.3 |
|  | O'Connor et al. 2022 | 1 (1.3%) | 1 (1.8%) | 1 |
| Pulmonary Collapse | Miller et al. 2024 | 83 (31.2%) | 78 (29.3%) | 0.71 |
| Pleural Effusion | O'Connor et al. 2022 | 1 (1.3%) | 1 (1.8%) | 1 |
| Acute Respiratory Distress Syndrome | Aryan et al. 2024 | 9 (1.2%) | 282 (1.9%) | 0.18 |
|  | Miller et al. 2024 | 5 (1.9%); acute respiratory failure | 7 (2.6%); acute respiratory failure | 0.77 |
| Atelectasis | Ba et al. 2015 | 1 (1.15%) | 3 (3.30%) | <0.05* |
|  | Maxwell et al. 2023 | 0 (0%) | 1 (3.8%) | 0.342 |
|  | Tanaka et al. 2020 | 0 (0%); requiring bronchoscopy | 5 (5%); requiring bronchoscopy | 0.588 |
| COPD Exacerbation | O'Connor et al. 2022 | 1 (1.3%) | 1 (1.8%) | 1 |
| Pulmonary Embolism | Aryan et al. 2024 | 5 (0.7%) | 263 (1.8%) | 0.02* |
|  | Maxwell et al. 2023 | 0 (0%) | 1 (3.8%) | 0.342 |
| Deep Vein Thrombosis | Aryan et al. 2024 | 22 (2.9%) | 512 (3.4%) | 0.47 |
| **Other, n (%)** | | | | |
| Reoperation | Aryan et al. 2024 | 21 (2.8%) | 465 (3.1%) | 0.66 |
|  | Marturano et al. 2023 | 2 (3.9%) | 14 (7.4%) | 1 |
|  | McElhinney et al. 2024 | 1 (4.3%); reoperation for postoperative bleeding | 0 (0%) | 0.41 |
|  | Maxwell et al. 2023 | 1 (4.3%) | 0 (0%) | 0.292 |
|  | Tung et al. 2022 | 0 (0%)† | 0 (0%)† | NR |
|  | Salan-Gomez et al. 2024 | 5 (6.1%); reoperation for bleeding | 6 (7.1%); reoperation for bleeding | 0.802 |
|  | Clemence et al. 2020 | 1 (4%); reoperation for bleeding/tamponade | 4 (4.3%); reoperation for bleeding/tamponade | 1 |
|  | Dokollari et al. 2023 | 1 (1.7%); reoperation for bleeding | 0 (0%); reoperation for bleeding | 0.392 |
| Readmission (30-day) | Marturano et al. 2023 | 3 (5.9%) | 7 (3.7%) | 0.88 |
|  | O'Connor et al. 2023 | 1 (7%); readmitted for pain control | 1 (8%); readmitted for pain control | >0.99 |
|  | Chidiac et al. 2024 | 2 (14.3%); reason for readmission was unrelated to pain | 2 (8.3%); reason for readmission was unrelated to pain | 0.55 |
|  | McElhinney et al. 2024 | 1 (4.3%); persistent pleural effusion† | 1 (6.7%); neutropenic sepsis following chemotherapy† | NR |
|  | Miller et al. 2024 | 30 (11.3%); at least 1 readmit through 30-days | 25 (9.4%); at least 1 readmit through 30-days | 0.57 |
|  | O'Connor et al. 2022 | 1 (1.3%); uncontrolled pain requiring readmission | 0 (0%); uncontrolled pain requiring readmission | 1 |
|  | Tung et al. 2022 | 0 (0%)† | 0 (0%)† | NR |
| ED Visit | Chen et al. 2023 | 0 (0%); return to ED for pain | 1 (2.6%); return to ED for pain | 0.9 |
|  | McElhinney et al. 2024 | 1 (4.3%) | 2 (13.3%) | 0.32 |
|  | Miller et al. 2024 | 17 (6.4%); at least 1 ED visit within 30-days | 18 (6.8%); at least 1 ED visit within 30-days | 1 |
| CI: confidence interval; COPD: chronic obstructive pulmonary disease; ED: emergency department; INC: intercostal nerve cryoablation; NR: not reported; SOC: standard of care | | | | |
| *p-value<0.05 for statistical comparison between cohorts; p-values reported based on statistical methods in referenced article | | | | |
| †No statistical analysis conducted by study authors to compare complication rates between cohorts | | | | |

**Supplemental Table 9.** Details of Neuropathic Symptoms and Resolution

| **Study** | **Neuropathic Symptom Description and Resolution** |
| --- | --- |
| Chen et al. 2023 | Two patients treated with INC experienced variable duration numbness and one patient experienced a burning sensation at the incision site. All numbness and burning sensation resolved by 6-month follow-up. |
| Choi et al. 2021 | One patient in the INC cohort (6%) reported mild lateral chest wall paresthesia at 6-month follow-up. No patients in the SOC cohort reported chest wall paresthesia at follow-up, but one patient reported difficulty sleeping due to chest wall pain at 1-month follow-up. |
| Fernandez et al. 2022 | Four patients in the INC cohort (10.5%) and six patients in the SOC cohort (28.6%) experienced chest wall paresthesia at short-term follow-up (0-3 months). No patients in the INC cohort and three patients in the SOC cohort (50%) experienced chest wall paresthesia at long-term follow-up (3-6 months). |
| Lau et al. 2021 | One patient in the INC cohort (1.6%) experienced allodynia at 6-month follow-up. The pain was not bothersome and required no further intervention through 10 months. |
| O’Connor et al. 2022 | One patient treated with INC experienced neuralgia 2 weeks post-discharge and required readmission for pain, with symptoms resolved by 3 months. The authors note that gabapentin, when taken as directed, is an important empiric agent for counteracting anticipated transient degrees of dysesthesias during nerve regeneration. |
| O’Connor et al. 2023 | Two patients treated with INC experienced neuralgia at 3 months following surgery with symptoms solved by 6-months. One of these patients had persistent numbness 1 year after surgery, but it was not bothersome. No patients in the INC cohort and two patients in the SOC cohort (17%) experienced postoperative chest wall neuralgia at 1 year. |
| Tung et al. 2022 | One patient in the SOC and one patient in the INC cohorts experienced neuralgia of the intercostal space and the ipsilateral anterior upper quadrant of the abdominal wall. Transient chest wall neuralgia resolved following treatment with one month of gabapentin therapy. |
| Weksler et al. 2024 | The Leeds Assessment of Neuropathic Symptoms and Signs (LANSS) was used two weeks after discharge, with higher LANSS scores and a higher proportion of patients with LANSS scores 12 or above with INC at two weeks post-procedure, the latter indicative of pain of neuropathic origin. |
| INC: intercostal nerve cryoablation; LANSS: Leeds Assessment of Neuropathic Symptoms and Signs; SOC: standard of care | |

**Supplemental Figures**
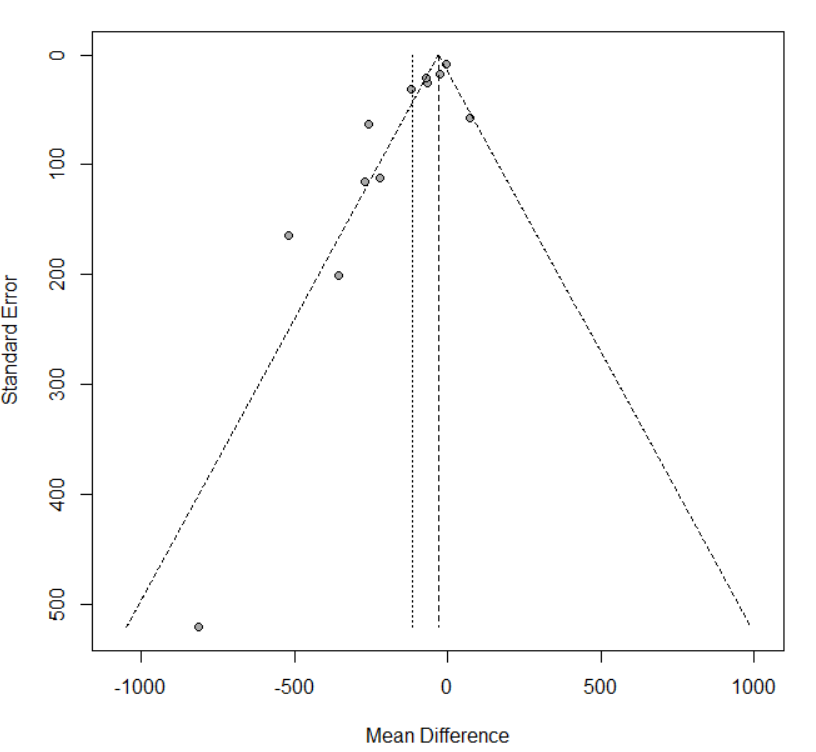


Test for Funnel Plot Asymmetry: z = 3.8176, p = 0.0001

Limit Estimate (as sei -> 0): b = 5.7757 (CI: -53.2652, 64.8165)

**Supplemental Figure 1.** Funnel plot and Egger’s regression test of inpatient opioid consumption assessing publication bias and small-study effects. CI: confidence interval.


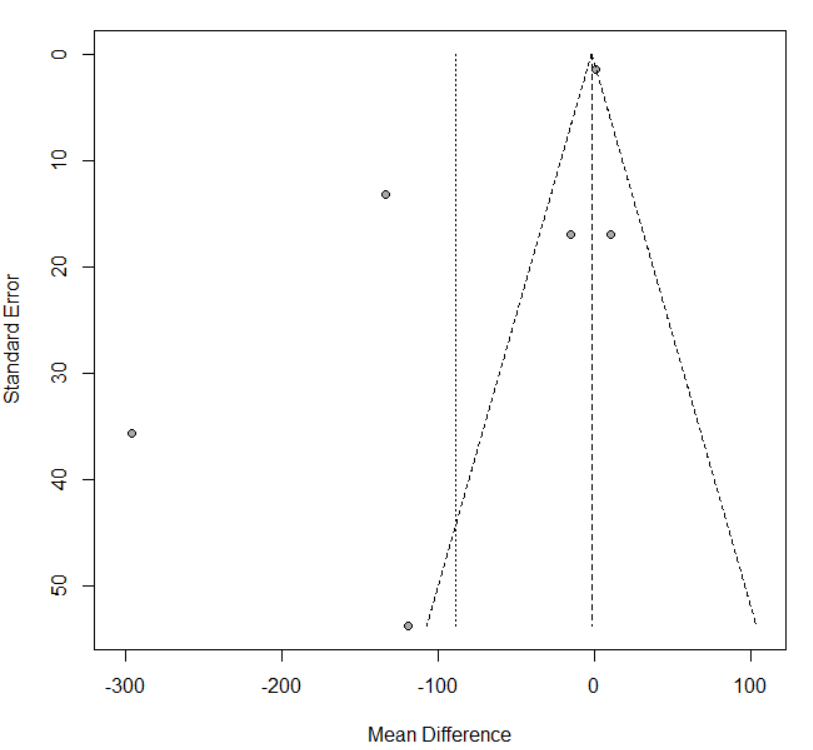


Test for Funnel Plot Asymmetry: z = 1.4106, p = 0.1584

Limit Estimate (as sei -> 0): b = 7.7581 (CI: -131.8074, 147.3237)

**Supplemental Figure 2.** Funnel plot and Egger’s regression test of outpatient opioid consumption assessing publication bias and small-study effects. CI: confidence interval.


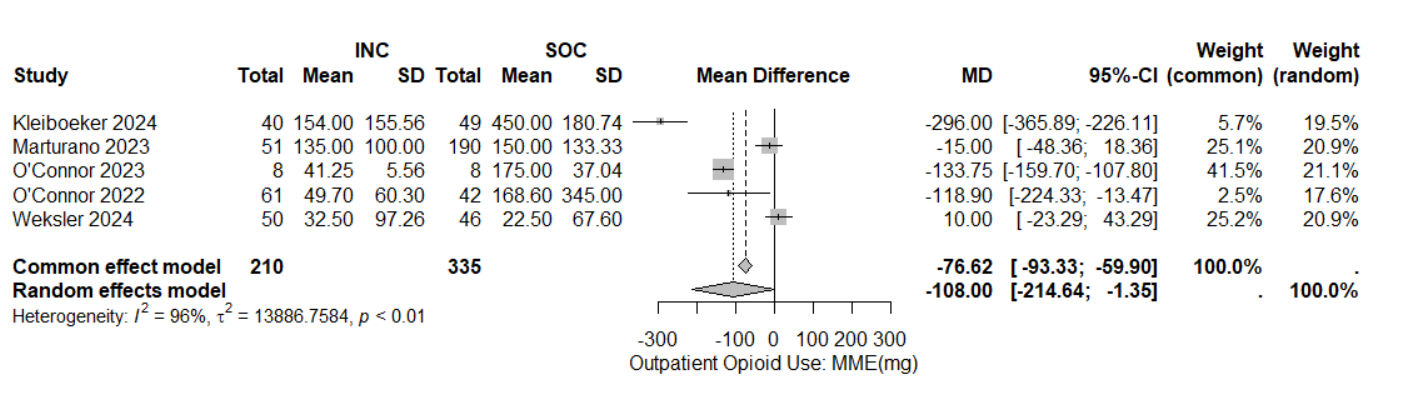


**Supplemental Figure 3.** Sensitivity analysis of the meta-analysis of outpatient opioid consumption (MME [mg]) to determine if the results of the meta-analysis were robust without database studies due to potentially overlapping patient populations with institutional studies. CI: confidence interval; INC: intercostal nerve cryoablation; MD: mean difference; MME: morphine milligram equivalents; SD: standard deviation; SOC: standard of care.


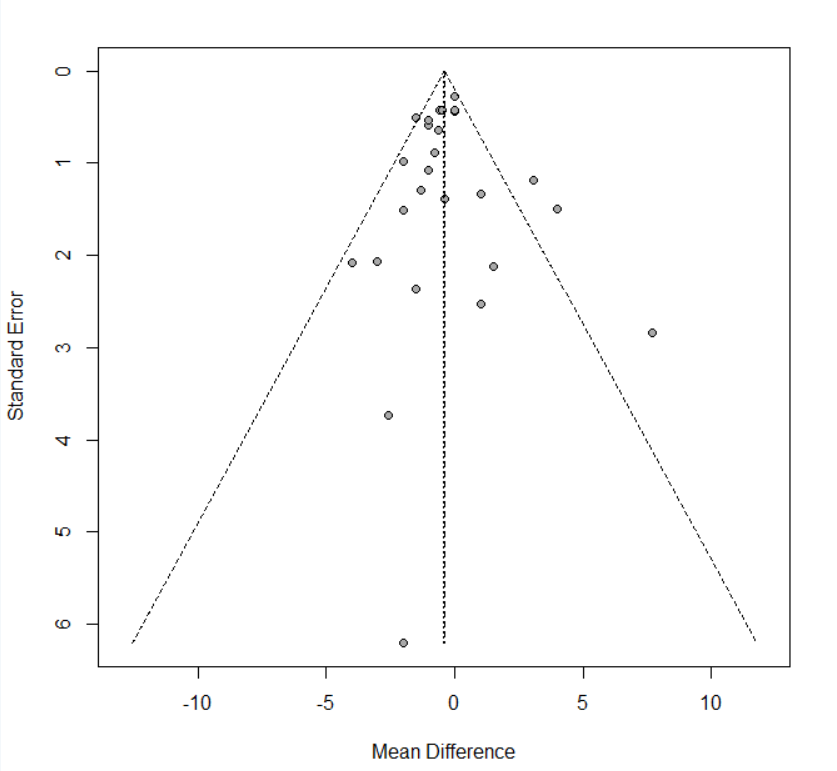


Test for Funnel Plot Asymmetry: z = 0.5889, p = 0.5559

Limit Estimate (as sei -> 0): b = -0.5988 (CI: -1.2513, 0.0538)

**Supplemental Figure 4.** Funnel plot and Egger’s regression test of hospital LOS assessing publication bias and small-study effects. CI: confidence interval; LOS: length of stay.


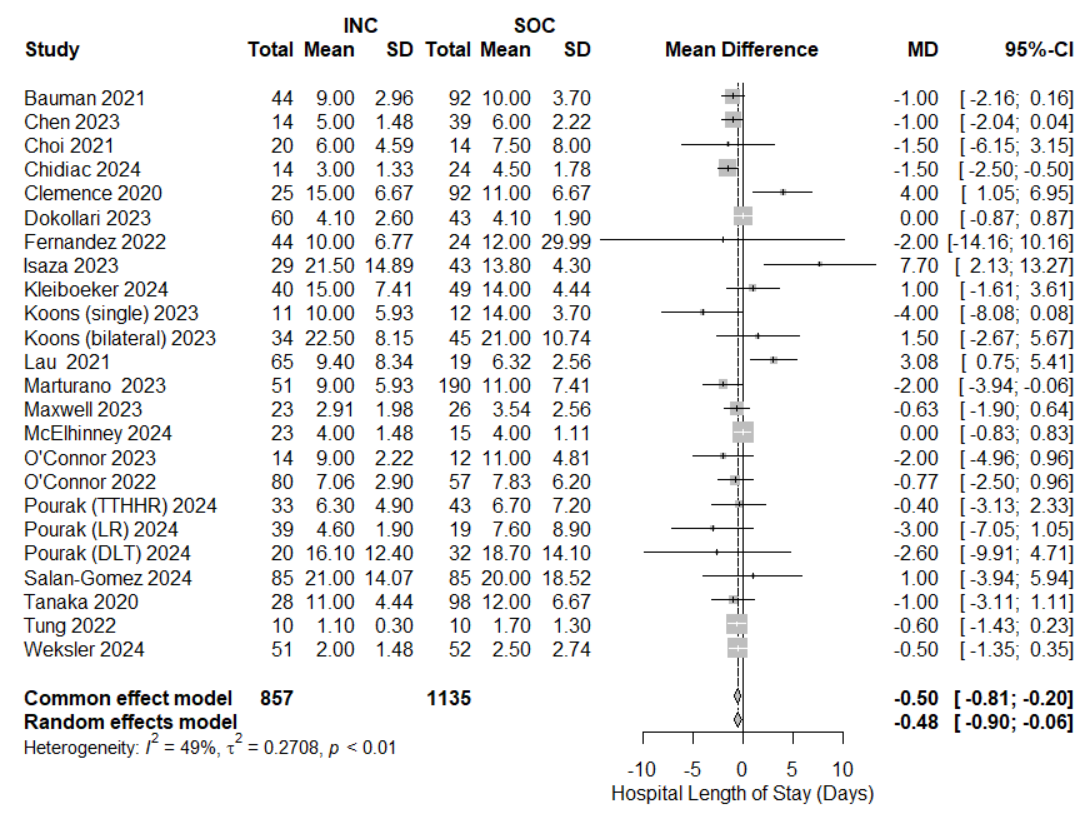


**Supplemental Figure 5.** Sensitivity analysis of the meta-analysis of hospital LOS to determine if the results of the meta-analysis were robust without database studies due to potentially overlapping patient populations with institutional studies. CI: confidence interval; DLT: double lung transplant; INC: intercostal nerve cryoablation; LOS: length of stay; LR: lung resection; MD: mean difference; SD: standard deviation; SOC: standard of care; TTHHR: transthoracic hiatal hernia repair.


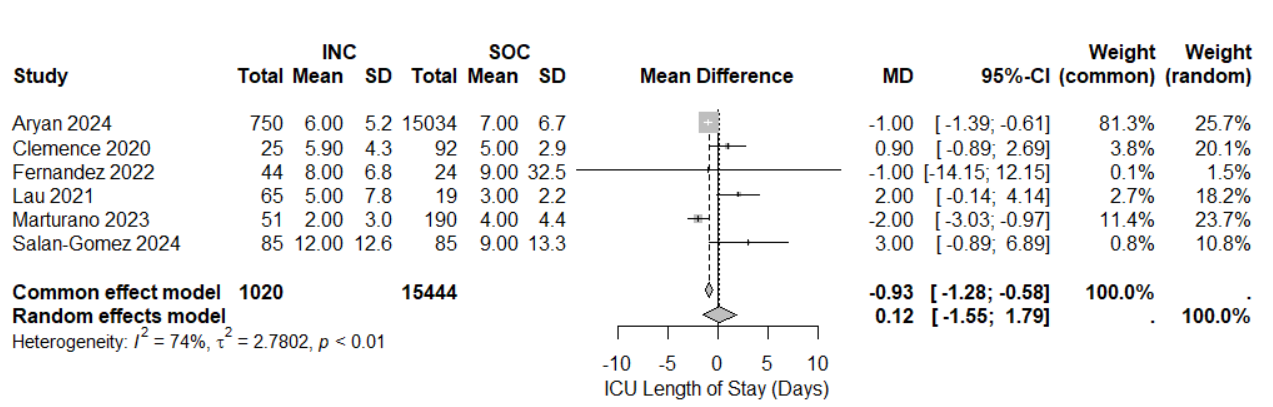


**Supplemental Figure 6.** Forest plot of ICU LOS. CI: confidence interval; ICU: intensive care unit; INC: intercostal nerve cryoablation; LOS: length of stay; MD: mean difference; SD: standard deviation; SOC: standard of care.
